# *Lactobacillus acidophilus* alleviate *Salmonella enterica* Serovar Typhimurium-induced murine inflammatory/oxidative responses via the p62-Keap1-Nrf2 signaling pathway and cecal microbiota

**DOI:** 10.3389/fmicb.2024.1483705

**Published:** 2025-01-16

**Authors:** Haihua Li, Xinyi Ma, Zhiyuan Shang, Xuejiao Liu, Jiayun Qiao

**Affiliations:** ^1^Tianjin Key Laboratory of Agricultural Animal Breeding and Healthy Husbandry, College of Animal Science and Veterinary Medicine, Tianjin Agricultural University, Tianjin, China; ^2^Tianjin Key Laboratory of Conservation and Utilization of Animal Diversity, College of Life Sciences, Tianjin Normal University, Tianjin, China; ^3^Xiaoyi Seventh Middle School, Xiaoyi, China

**Keywords:** *Lactobacillus acidophilus*, *Salmonella* Typhimurium, inflammatory responses, oxidative responses, p62-Keap1-Nrf2 signaling pathway, cecal microbiota

## Abstract

**Background:**

*Salmonella enterica* Serovar Typhimurium (*S*. Typhimurium) infection can cause inflammation and oxidative stress in the body, leading to gastroenteritis, fever and other diseases in humans and animals. More and more studies have emphasized the broad prospects of probiotics in improving inflammation and oxidative stress, but the ability and mechanism of *Lactobacillus acidophilus* (LA) to alleviate the inflammatory/oxidative reaction caused by pathogens are still unclear.

**Methods and results:**

In this study, we treated the mice with LA for 14 days, infected them with *S*. Typhimurium for 24 h, and sacrificed the mice to collect samples. We found that the early intervention of LA alleviated the pathological injury and reversed the down-regulation of the duodenal and hepatic tight junction protein mRNA levels caused by *S*. Typhimurium infection. Compared with *S*. Typhimurium group, LA early intervention increased the expression of antioxidant enzymes, but decreased the levels of serum malondialdehyde (MDA), interleukin-8 and tumor necrosis factor-α (TNF-α). Additionally, LA early intervention significantly increased *Nrf2* mRNA expression in the liver and decreased *Keap1* mRNA expression in the duodenum compared to the *S*. Typhimurium group. Furthermore, early LA treatment reduced the abundance of *Bacteroides acidificiens*, increased the abundance of *Akkermansia*, and alleviated the decrease in SCFAs levels in the cecum of *S*. Typhimurium-infected mice. Spearman correlation analysis showed that there was a certain correlation between cecal flora and serum indicators and short chain fatty acids.

**Conclusion:**

Taken together, the results indicate that LA early intervention may alleviates *S*. Typhimurium-induced inflammation and oxidative responses in mice by activating the p62-Keap1-Nrf2 signaling pathway and regulating the gut microbial community.

**Significance and impact of the study:**

Exploring the ability of LA to resist animal oxidative stress and microflora regulation caused by pathogenic microbes, so as to provide more options for developing healthy disease-resistant feed additives.

## 1 Introduction

*Salmonella* is a pathogenic Gram-negative bacterium that exists in humans and animals, causing gastroenteritis, diarrhea, and even life-threatening conditions (Hernandez et al., 2003; Wan et al., 2019). *Salmonella* is able to overcome, manipulate, and even exploit host immune defenses to facilitate its replication and systemic spread within the host and establish a persistent infection, and the pathogenic substances of *Salmonella* mainly include flagellin, lipopolysaccharide (LPS), and the metabolites hydrogen sulfide (H_2_S), while some *Salmonella* can also produce enterotoxins (Luca et al., 2019; Tang et al., 2016; Eaves-Pyles et al., 2001; Ding et al., 2004). Pattern receptor recognition of flagellin and LPS induces phosphorylation and the degradation of inhibitor kappa B kinaseα (IKKα) protein, and subsequently prompt nuclear factor-kappaB (NF-κB) to enter the nucleus. This mediates the expression of multiple pro-inflammatory factor genes, disrupts tight junctions, and triggers tissue barrier dysfunction, inducing an inflammatory response (Gewirtz et al., 2001; Mazgaeen and Gurung, 2020; Kim et al., 2019; Li et al., 2016). In addition, Flagellin bind and activate TLR5 receptors and induce oxidative stress in mice (Liaudet et al., 2002). LPS up-regulates the expression of related oxidases in macrophages and produces a large amount of reactive oxygen species (ROS) (Wu et al., 2020). ROS promotes phosphorylation of protein kinase B (Akt) in mouse macrophages; this plays a key role in activating NF-κB signaling and further aggravate the inflammatory response (Wang et al., 2022; Komatsu et al., 2018). *Salmonella* bacteria can produce H_2_S by degrade sulfur amino acids, which decrease mitochondrial oxygen consumption and causes overexpression of inflammatory factors (Luca et al., 2019; Shibuya et al., 2009). Furthermore, *Salmonella*’s virulence proteins disrupt mitochondrial structure, causing oxide buildup (Herrero-Fresno and Olsen, 2018). Oxidative stress is closely linked to the inflammatory response. Therefore, reducing oxidative stress is a potential method to reduce the inflammatory damage caused by *Salmonella* infection. MDA is produced by the peroxidation of polyunsaturated fatty acids in the body and has been used as an indicator of oxidative stress (Ho et al., 2013). Glutathione peroxidase and sodium sulfate are antioxidants and the first line of defense for the body to deal with oxidative stress. SODs catalyze the disproportionation reaction to promote the conversion of superoxide anion (O_2_-) into molecular oxygen and H_2_O_2_, while GSH-Px facilitates the reduction of H_2_O_2_ to H_2_O, thereby achieving stability and structural protection of the intra-cellular environment (Valko et al., 2006; Blake et al., 1987). Heme oxygenase-1 (HO-1) is a stress-induced enzyme important for antioxidant defense and apoptosis (Bindu et al., 2011). The metabolites of HO-1, including carbon monoxide, biliverdin, and ferrous ions, inhibit the expression of NF-κB and its target genes, thereby exerting anti-inflammatory effects (Lee and Chau, 2002; Kang et al., 2019; Liu et al., 2008).

NF-E2-related factor 2 (Nrf2) plays a critical role as a transcriptional activator that regulates metabolic processes, immune responses, and is associated with cell proliferation (Silva-Islas and Maldonado, 2018). Under normal conditions, Nrf2 levels are maintained at low levels, which binds to kelch-like ECH-associated protein-1 (Keap1) and relies on Cullin 3 (Cul3) to mediate its ubiquitination and degradation (Sun et al., 2016). Under stress conditions, cysteine residues of Keap1 are modified, prompting the separation of Nrf2 from the Keap1/Nrf2 complex. Activated Nrf2 is transferred into the nucleus, where it binds to Maf protein to form a heterodimer and then recognizes and binds to the anti-oxidative response element (ARE), mediating the expression of downstream anti-oxidant and anti-inflammatory genes (Suzuki et al., 2013). Recent studies have demonstrated that selective autophagy is able to eliminate aggregated proteins, diseased and damaged organelles, and invasive pathogens via the ubiquitin signaling pathway (Keller et al., 2020; Khaminets et al., 2016; Ashrafi and Schwarz, 2013). P62/SQSTM1 (p62) is an autophagy receptor protein that when phosphorylated competes with Nrf2 for binding sites on Keap1 under stress conditions, inhibiting the formation of the Keap1/Nrf2 complex and linking the Keap1-Nrf2 signaling pathway to selective autophagy as a host defense mechanism to alleviate inflammation and oxidative stress (Ichimura et al., 2013).

The gut microbiota is an important component of the human immune system. On the one hand, the beneficial gut microbiota competes with invading pathogens for nutrients and provides colonization resistance, protecting the host from pathogenic microorganisms (Stecher et al., 2013); on the other hand, it promotes the maturation of the immune system and maintains the integrity of intestinal epithelial cells, and produce gut health-promoting metabolites such as SCFAs (Sommer and Bäckhed, 2013; Kayama et al., 2020). Invasion of exogenous pathogens will disrupt the microbial community, destroy colonization resistance, and lead to intestinal dysbiosis (Ducarmon et al., 2019). Intestinal dysbiosis increases the permeability of the intestinal barrier, leading to the translocation of pathogenic microorganisms and leakage of intestinal con-tents, triggering inflammation and oxidative stress, and ultimately threatening human health (Meng et al., 2018). Therefore, restoring or maintaining gut microbial balance is an effective strategy to alleviate inflammation and oxidative stress.

Most *Lactobacillus* are widely used as probiotic with anti-inflammatory, antioxidant, and intestinal flora-regulating capacities (Jiang et al., 2018; Huang et al., 2020). As previously reported, *Lactobacillus acidophilus* (LA) has the properties of acid and bile tolerance, competitive colonization, and the ability to use carbohydrates and other sources of nutrients in the intestine (Azcarate-Peril et al., 2004; Pfeiler et al., 2007; Goh and Klaenhammer, 2014). These properties of LA help it adapts to extreme environments. In an *in vitro* study, Lépine et al. (2018) demonstrated that LA W37 protects against *Salmonella* Typhimurium (*S*. Typhimurium)-induced disruption of Caco-2 cell integrity by regulating the expression of tight-junction related genes and the secretion of cytokines. However, it is unclear whether LA remains effective in protecting against *S*. Typhimurium-induced inflammation and oxidative responses in animals. Therefore, in this study, a pathogenic model was established using *S*. Typhimurium-infected mice *in vivo,* with the p62-Keap1-Nrf2 signaling pathway and cecum microbiota used as entry points to investigate the role and mechanism of LA in *S*. Typhimurium-induced inflammation and oxidative responses in mice.

## 2 Materials and methods

### 2.1 Ethics statement

The animal use protocol for this research was approved by the animal care and use committee of Tianjin Agricultural University.

### 2.2 Bacterial strains and culture conditions

LA was isolated from the feces of healthy piglets and was identified based on observation of colony morphology, Gram staining, and sequence comparison of the PCR products of 16S rDNA. The LA was grown in MRS (De Man, Rogosaand Sharpe) medium at 37°C under anaerobic environment. Culture solution of the strain was centrifuged at 3,000 × g for 10 min at 4°C. Bacterial powder was acquired according to the treatment in a vacuum freeze-drying machine (Tofflon, Shanghai, China), and there are 5 × 10^10^ CFU/g LA in freeze-drying powder.

*Salmonella* was isolated from the intestinal contents of sick piglets. The intestinal contents of diseased pigs were firstly isolated and cultivated using the standard plate culture method under aerobic conditions. Purified single colonies were then inoculated onto MacConkey and Triple Sugar Iron Agar for identification and further culture, respectively. The *Salmonella* was identified as *S*. Typhimurium by bacterial isolation culture, microscopic examination, biochemical test and PCR. The PCR was used to amplify 16S rRNA gene using primers 16S-F 5′-GTTACCCGCAGAAGAAGCAC-3′, and 16S-R 5′-CCCCCCTCTACAAGACTCAA-3′. It is now preserved by the Laboratory of the College of Animal Science and Veterinary Medicine at Tianjin Agricultural University.

### 2.3 Animals and experimental design

Sixty-four 6- to 8-week-old specific pathogen-free (SPF) male Kunming mice were selected. After a three-day period of adaptation, mice were randomly divided into four groups (CK group, ST group, LA group, and LA + ST group), with 16 replicates in each group. The following treatments were performed: (1) Mice in the CK group were given saline by oral gavage on days 1–15; (2) mice in the ST group were given saline by oral gavage on days 1–14 and 3.2 × 10^8^ CFU/mL *S*. Typhimurium on day 15; (3) mice in the LA group were given 1 × 10^7^ CFU/mL LA by oral gavage on days 1–15; (4) mice in the LA + ST group were given 1 × 10^7^ CFU/mL LA by oral gavage on days 1–14 and 3.2 × 10^8^ CFU/ mL *S*. Typhimurium on day 15. Mice were treated once daily, and the volume was 0.2 mL.

### 2.4 Sample collection and processing

After 24 h of *S*. Typhimurium treatment, six mice of similar body weight were selected in each group; blood samples were collected from the inner canthus of the eyes, and the serum was separated. The collected serum was stored at −80°C to be used in determining the levels of superoxide dismutase (SOD), glutathione peroxidase (GSH-Px), malondialdehyde (MDA), tumor necrosis factor-α (TNF-α), and interleukin-8 (IL-8). After obtaining blood samples, the mice were sacrificed using the cervical dislocation method. Duodenal and liver tissues were collected under sterile conditions. One part was fixed in 4% paraformaldehyde to be used to observe the morphological structure of the tissues, while the other part was immediately immersed in liquid nitrogen and then transferred to −80°C for storage to be used to detect the expression of the target genes. The cecum contents were collected for the analysis of changes in the microbial community and for the detection of short-chain fatty acids (SCFAs) levels.

### 2.5 Histological examination

The liver and duodenum of mice fixed with paraformaldehyde were dehydrated by gradient alcohol, transparent, paraffin embedded, sectioned (3–5 μm), stained with hematoxylin–eosin (H&E), and finally sealed by neutral balsam. The prepared sections were observed and photo-graphed using an ECHO Revolve Hybrid microscope. The experimental procedure followed the double-blind principle. Through pathological sections, we found that the main characteristics of duodenum were inflammatory cell infiltration, epithelial cell separation from lamina propria and so on. Therefore, this study refers to the scoring methods of duodenum in other studies, establishes the standards that can be used to quantify the sections of this study, and quantitatively scores the obtained sections (Supplementary Table S1) (Chiu et al., 1970; Kamel et al., 1988). Similarly, the main characteristics of the liver are inflammatory cell infiltration, liver cell punctate necrosis and so on. This study refers to the scoring methods of liver in other studies, establishes the standards that can be used to quantify the sections of this study, and quantifies the sections (Supplementary Table S2) (Suzuki et al., 1993). The sections of 6 mice in each group were selected, and 3–4 parts of each section were selected for scoring.

### 2.6 Detection of serum indicators

SOD was detected by xanthine oxidase method (A001-3-2), GSH-Px was detected by visible light colorimetric method (A005-1-2), and MDA content was detected by TBA colorimetric method (A003-1-2). The iNOS was detected by an iNOS assay kit (H372-1-2). The kits were purchased from Jiancheng Biological Engineering Institute (Nanjing, China). The TNF-α (H052-1-2) and IL-8 (H008-1-2) levels in the serum were determined using ELISA kits (R&D Systems, Minneapolis, MN). The detection process referred to our previous study (Sun et al., 2020).

### 2.7 Quantitative real-time PCR

Total tissue RNA was extracted using RNAzol reagent (Yanshun Biotechnology Co., Ltd., Shenzhen, China). The quality and concentration of RNA were assessed using a NanoDrop spectrophotometer. RNA was reverse-transcribed to cDNA in strict accordance with the instructions of the All-in-OneTM First cDNA Synthesis kit (GeneCopoeia, Rockville, United States). Then, the qPCR reaction system was prepared according to the instructions of the All-in-One^™^ qPCR Mix kit (Gene-Copoeia, Rockville, United States). The Ct values of the target genes were obtained by LightCycler ^®^480 Real-Time PCR System (Roche), and the relative expression levels of those genes were calculated by the 2^−ΔΔCt^ method using glyceraldehyde-3-phosphate dehydrogenase (GAPDH) as the reference gene. The qPCR amplification conditions were as follows: 95°C pre-denaturation for 10 min, 95°C for 15 s, 56°C/62°C (this temperature was determined by the primer annealing temperature) for 20 s, 72°C for 20 s, for 40 cycles. Primer 5.0 was used to design the p62 and Nrf2 specific primers after obtaining the complete sequence of the target gene from NCBI. The specific sequences of zonula occludens-1 (*ZO-1*), *occludin*, *claudin-1*, *claudin-8*, *SOD1*, *SOD2*, heme oxygenase-1 (*HO-1*), *GSH-Px1*, *Keap1* and *GAPDH* were described in previous reports and were synthesized by Sangon Biotech Co., Ltd. (Shanghai, China) (Table 1) (Ruan et al., 2014; Li et al., 2018; Sassi et al., 2020; Li et al., 2019; Cheng et al., 2019; Yang et al., 2018; Shen et al., 2017).

**Table 1:** Primer sequences information.

Genes	Primers sequences/(5′→3′)	Product length/bp	Annealing temperature/°C	GenBank accession number
*SOD1*	F: AACCAGTTGTGTTGTGAGGAC R: CCACCATGTTTCTTAGAGTGAGG	139	56	NM_011434.2
*SOD2*	F: ACGCCACCGAGGAGAAGTACC R: CGCTTGATAGCCTCCAGCAACTC	181	62	NM_013671.3
*HO-1*	F: CAAGGAGGTACACATCCAAGCC R: TACAAGGAAGCCATCACCAGCT	102	56	NM_010442.2
*GSH-Px1*	F: GAAGTGCGAAGTGAATGG R: TGTCGATGGTACGAAAGC	224	56	NM_001329528.1
*p62*	F: CAACTGTTCAGGAGGAGACGA R: CTGGTGGCAGATGTGGGTA	179	62	XM_036156414.1
*Keap1*	F: CTGGTATCTGAAACCCGTCTA R: TGGCTTCTAATGCCCTGA	117	56	NM_001110307.1
*Nrf*2	F: CAGTGCTCCTATGCGTGAA R: GCGGCTTGAATGTTTGTC	109	56	NM_010902.4
*ZO-1*	F: GGGAGGGTCAAATGAAGACA R: GGCATTCCTGCTGGTTACAT	145	56	XM_036152895.1
*Occludin*	F: CCTTCTGCTTCATCGCTTCCTTA R: CGTCGGGTTCACTCCCATTAT	164	62	NM_001360538.1
*Claudin-1*	F: GAGTCTCCGGTGCATCATTT R: CAGCTTGCTAGGGAACTTGG	143	56	NM_001379248.1
*Claudin-8*	F: GTGCTGCGTCCGTCTTGGCT R: TCGTCCCCCGTGCATCTGGT	79	62	NM_018778.3
*GAPDH*	F: GCACAGTCAAGGCCGAGAAT R: GCCTTCTCCATGGTGGTGAA	151	56/62	XM_036165840.1

F shows forward primer; R shows reverse primer.

### 2.8 Western blotting

Mouse liver and duodenal tissues were fully homogenized in 1 mL of lysis buffer with protease and phosphatase inhibitors to obtain total protein. The concentration of extracted protein was estimated using a bicinchoninic acid (BCA) protein assay kit (Pierce Chemical Co., Rockford, IL, United States). The target protein was separated using sodium dodecyl sulfate-polyacrylamide gel electrophoresis (SDS-PAGE gel electrophoresis), and then transferred onto a polyvinylidene fluoride (PVDF) membrane. Add 5% skimmed milk and incubate at room temperature for 90 min, then incubate overnight with primary antibodies at 4°C to bind to antigens and antibodies. The primary antibodies were as follows: anti-GAPDH (Abways, AB0037, rabbit monoclonal IgG), anti-AKT1 + AKT2 + AKT3 antibody (Abcam, ab179463, rabbit monoclonal IgG), anti-NF-κB p65 anti-body (Abcam, ab16502, rabbit monoclonal IgG), and anti-IKKα antibody (Abcam, ab32041, rabbit monoclonal IgG). Then, the membrane was incubated with anti-rabbit (Abcam, ab6721) horseradish peroxidase (HRP) for 90 min. After several washing steps, the protein signal was detected using ECL chemiluminescence reagent. The ImageJ software was used to quantify the bands. There were 6 mice in each group, and each mouse was tested three times.

### 2.9 16S rDNA amplicon sequencing and microbiota analysis

The cecal contents of three mice in each group were randomly selected for 16 s DNA amplocon sequencing. Cecal genomic DNA was extracted from cecal contents using the cetyltrimethyl ammonium bromide (CTAB) method. The 16S rDNA primer sequence with barcode (341F: 5′-CCTAYGGGRBGCASCAG-3′; 806R: 5′-GGACTACNNGGGTATCTAAT-3′), Phusion^®^ High-Fidelity PCR Master Mix with GC Buffer (New England Biolabs, Ipswich, MA) and high-fidelity enzymes were used to amplify the 16S rDNA V3–V4 region of the cecal contents. The PCR product was determined by 2% agarose gel electrophoresis, and then purified with Qigen Gel Extraction Kit (Qiagen, Hilden, Germany). TruSeq using PCR free technology^®^A DNA sample preprocessing kit was established and a library was established. After being quantified by Qubit and Q-PCR, the constructed library was sequenced on an Illumina MiSeq platform (Illumina). The above process was completed by Beijing Novogene Biology Information Technology Co., Ltd. (Beijing, China).

The PE-reads after removing barcode and primer sequences were spliced into one sequence by Flash (V1.2.7) to obtain raw tags (Magoč and Salzberg, 2011). Raw tags filtering and analysis were performed by Qiime 1.9.1 to obtain clean tags (Caporaso et al., 2010). Clean tags went through quality control and removal of chimeric sequences to obtain effective tags (Caporaso et al., 2010; Haas et al., 2011). The sequences were clustered using Uparse software (v7.0.1001), and those with similarities greater than 97% were grouped into the same operational taxonomic unit (OTU) (Haas et al., 2011). Each OTU sequence was annotated and analyzed using the Mothur method and the SSUrRNA database from SILVA132 (Wang et al., 2007; Quast et al., 2013). Qiime software (v1.9.1) and R soft-ware (Version 2.15.3) were used to calculate α-diversity (including indicators such as Shannon and Chao1 indices) and β-diversity, and then the results were visualized. Spearman correlation analysis was used to explore the relationship between oxidative indicators, inflammatory indicators, and microbial communities. Select meaningful indicators to display according to the Spear-man correlation results.

### 2.10 Measurement of SCFA concentrations

The contents of acetate, propionate, butyrate, and total short-chain fatty acids (SCFAs) in mouse cecum contents were determined using an Agilent 7890B gas chromatograph system and an Agilent 5977B mass spectrometer according to the method of Zheng et al. (2013).

### 2.11 Statistical analysis

The data are expressed as mean ± standard error (SEM). The statistical analysis of data was performed by IBM SPSS version 26.0 (Armonk, NY, United States). Significant differences were analyzed using one-way analysis of variance (ANOVA) with Tukey’s post-hoc tests or by Welch’s ANOVA with Dunnett’s T3 post-hoc tests. Significant differences in histopathological scores were compared by Mann–Whitney U tests. *p* < 0.05 was considered to be a significant difference. The histograms were drawn by GraphPad Prism V.7.0 (GraphPad Software, San Diego, CA, United States).

## 3 Results

### 3.1 Effect of LA on liver and duodenal damages in *Salmonella* Typhimurium-induced mice

**Figure 1:**
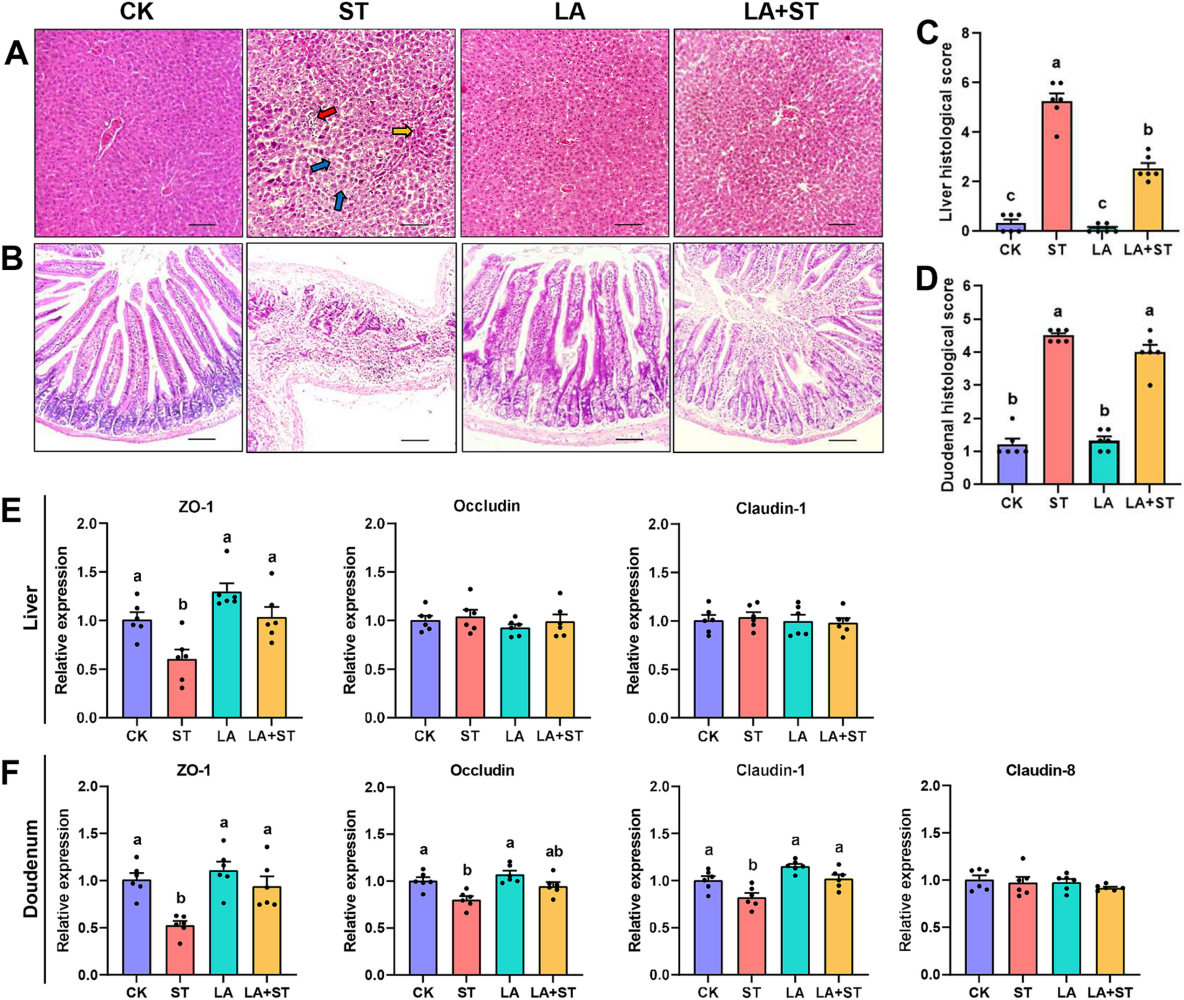
Effect of LA on liver and duodenal damages in *S*. Typhimurium-induced mice. **(A)** Images of hematoxylin and eosin-stained liver tissue sections and **(B)** histological scores (six mice per group); **(C)** Images of hematoxylin and eosin-stained duodenal tissue sections and **(D)** histological scores (6 mice per group); **(E)** The mRNA expression levels of tight-junction related genes in the liver were detected by qPCR (6 mice per group); **(F)** Using fluorescence quantitative PCR technology (6 mice in each group) to study the mRNA expression of tight junction related genes in the duodenum. Data are expressed as mean ± SEM. Different lowercase letters on columns indicate a significant difference at the 0.05 level.

In the pre-test, we found that the liver and duodenum of mice infected with *S*. Typhimurium had the most obvious changes after 24 h of dissection. Therefore, liver and duodenum were selected for this study to understand the effects of LA and *S*. Typhimurium on the morphological structure of the liver and duodenum in mice, histological sections were observed and scored in this study. As shown in Figure 1A, hepatocytes in the CK and LA groups were neatly arranged and tightly structured. Localized lymphocytic infiltration was observed in the LA group compared to the CK group. In the ST group, the hepatic cords were loosely arranged; the cytoplasm of hepatocytes was lax (shown by blue arrows); some nuclei exhibited pyknoses (shown by yellow arrows in the figure) or disappeared; and the lymphocytes were aggregated and showed punctate necrosis (shown by red arrows). The liver tissue in the LA + ST group showed disorganized arrangement of hepatic cords, cytoplasmic laxity of some hepatocytes around the central veins, and an increase of the hepatic sinusoidal area. Liver histological scores were significantly increased in the ST group compared with the CK group (*p* < 0.05; Figure 1C). Moreover, compared to the ST group, mice in the LA + ST group exhibited a significantly lower liver histological score (*p* < 0.05; Figure 1C). As shown in Figure 1B, mice in the CK and LA groups had intact duodenal villi and only a small amount of inflammatory cell infiltration in the lamina propria. In the ST group, the normal structures of villi and crypts basically disappeared; lymphocytes had infiltrated, and severe ulceration occurred in the lamina propria. Mice in the LA + ST group had ruptured and fused villi and increased inflammatory cells in the lamina propria. *S*. Typhimurium treatment significantly increased the duodenal histological score (*p* < 0.05; Figure 1D), whereas duodenal histological scores were lower in the LA + ST group than in the ST group, although the difference was not significant (*p* > 0.05; Figure 1D).

To investigate the effects of LA and *S*. Typhimurium on the structure and function of the liver and duodenal barrier, the mRNA expression levels of tight junction protein (*ZO-1*, *occludin*, *claudin-1* and so on) were examined in this study. As shown in Figures 1E,F compared with the CK group, *S*. Typhimurium infection significantly decreased the mRNA expression levels of *ZO-1* in the liver (*p* < 0.05), and the mRNA expression levels of *ZO-1*, *claudin-1* and *occludin* in the duodenum (*p* < 0.05). Although LA treatment alone has no significant changes in these proteins (*p* > 0.05), an increasing trend can be observed in the mRNA expression levels of *ZO-1* in the liver. Compared with the ST group, LA early intervention significantly increased the mRNA expression levels of *ZO-1* and *claudin-1* in the duodenum, as well as the mRNA expression levels of *ZO-1* in the liver (*p* < 0.05). The above results suggested that early intervention of LA can alleviate *S*. Typhimurium-induced liver and duodenal damage to a certain extent.

### 3.2 Effect of LA on inflammatory response in *Salmonella* Typhimurium-induced mice

**Figure 2:**
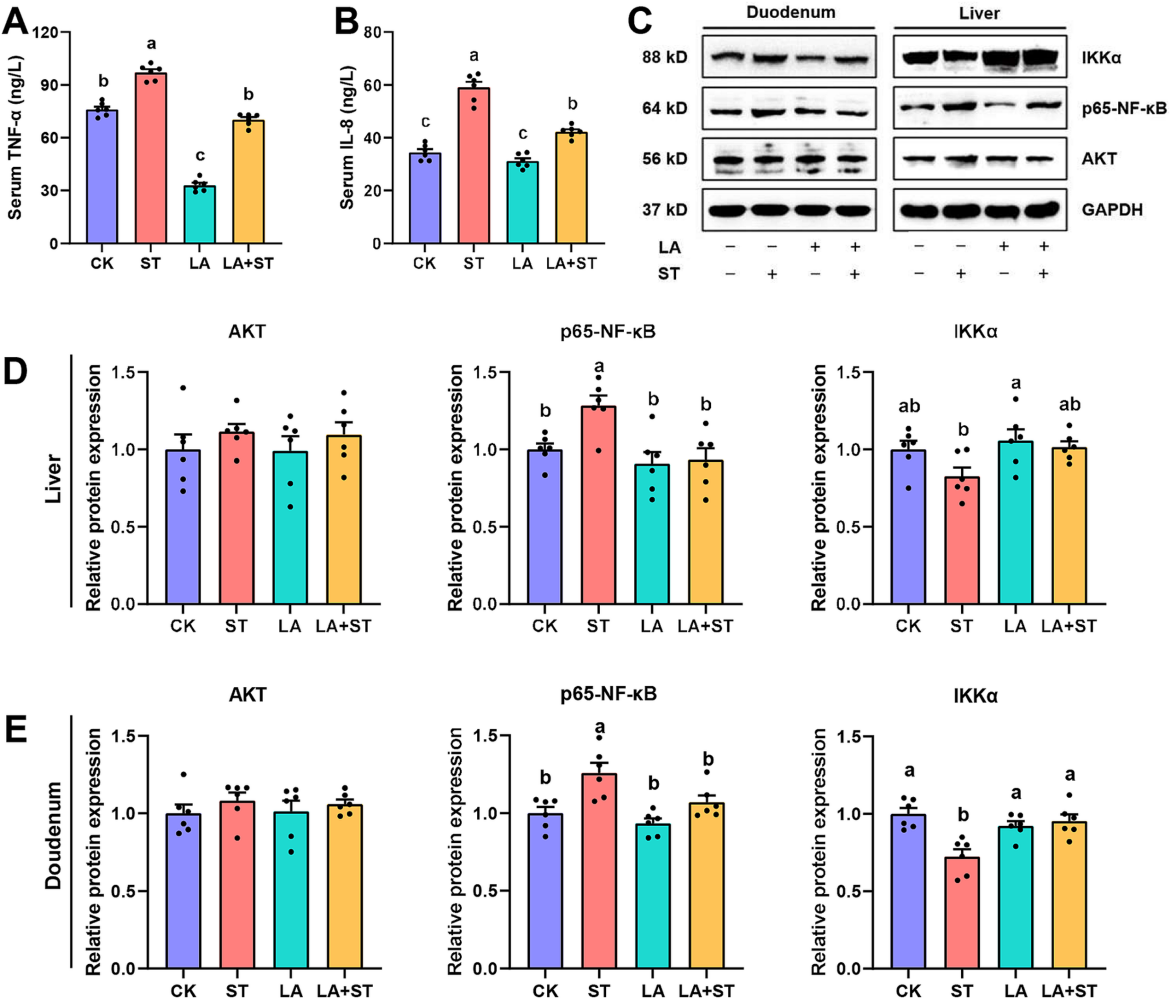
Effect of LA on the inflammatory response in *S*. Typhimurium-induced mice. The contents of TNF-α **(A)** and IL-8 **(B)** in the serum were detected using ELISA (6 mice per group). The protein expression levels of Akt, IKKα, p65-NF-κB in the liver **(C,D)** and duodenum **(C,E)** were detected using western blotting (6 mice per group). Data are expressed as mean ± SEM. Different lowercase letters on columns indicate a significant difference at the 0.05 level.

To understand the effects of LA and *S*. Typhimurium on inflammatory responses in mice, this study examined serum, liver, and duodenal inflammatory response markers. As shown in Figure 2, compared the mice in the ST group with those in the CK group, TNF-α and IL-8 levels in the serum were significantly increased, while in the liver and duodenum, p65-NF-κB protein expression was significantly increased and IKKα protein expression was only significantly decreased in duodenum (*p* < 0.05). However, these abnormal changes were reversed in mice with LA early intervention. The protein expression levels of Akt did not significantly different in the CK, ST, LA and LA + ST groups (*p* > 0.05). Taken together, the results indicate that early intervention of LA can help to alleviate *S*. Typhimurium-induced inflammatory responses.

### 3.3 Effect of LA on oxidative response in *Salmonella* Typhimurium-induced mice

**Figure 3:**
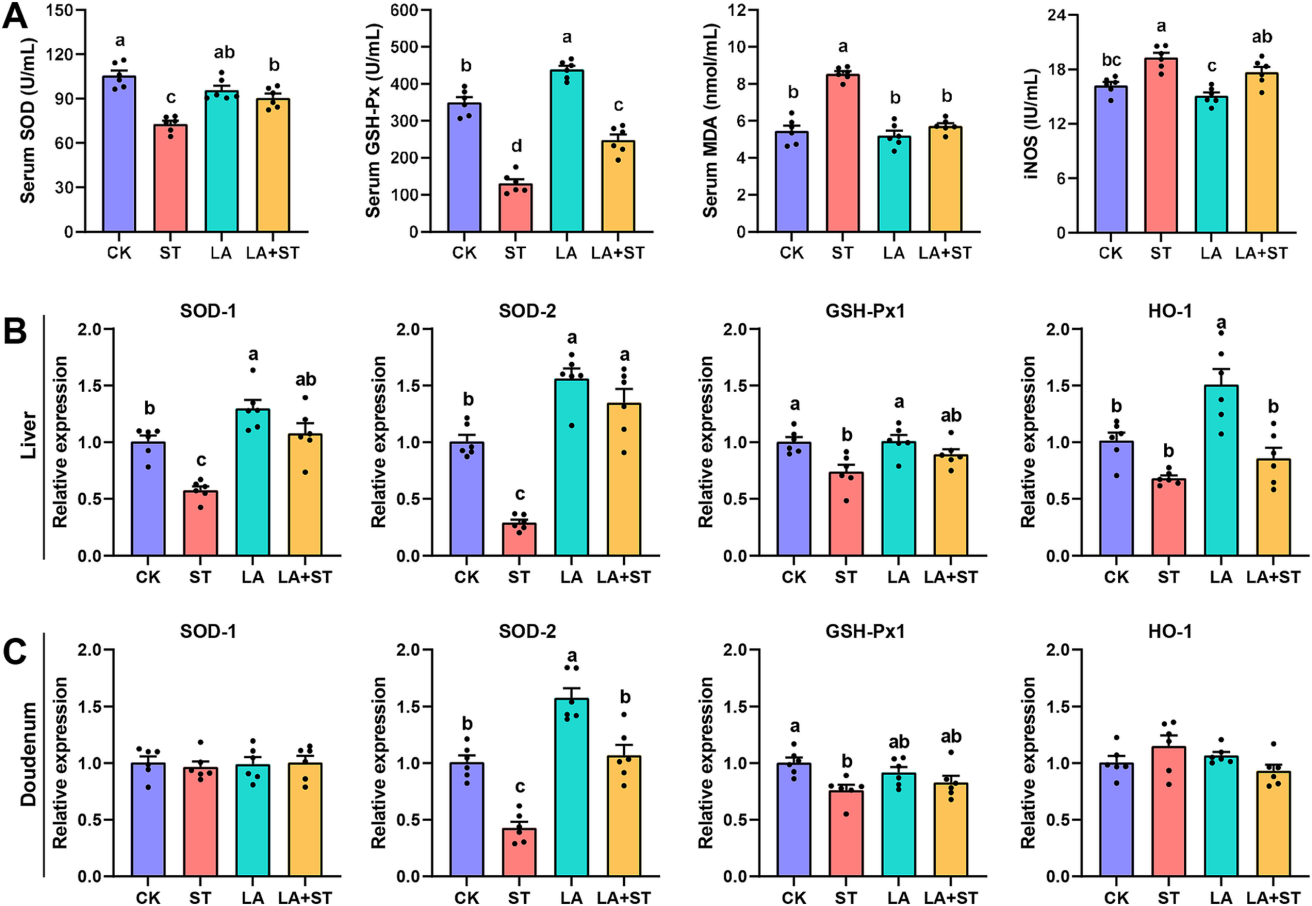
Effect of LA on oxidative response in *S*. Typhimurium-induced mice. **(A)** The contents of SOD, GSH-Px, MDA and iNOS in the serum were detected (6 mice per group). The mRNA expression levels of *SOD1*, *SOD2*, *GSH-Px1*, and *HO-1* in the liver **(B)** and duodenum **(C)** were detected using qPCR (6 mice per group). Data are expressed as mean ± SEM. Different lowercase letters on columns indicate a significant difference at the 0.05 level.

To understand the effects of LA and *S*. Typhimurium on oxidative response in mice, this study examined serum, liver, and duodenal oxidative response markers. As shown in Figure 3A, LA treatment alone significantly elevated GSH-Px levels in the serum (*p* < 0.05). *S*. Typhimurium infection significantly decreased SOD and GSH-Px levels and significantly increased MDA and iNOS levels in the serum compared to the CK group (*p* < 0.05), while the changes in SOD, GSH-Px and MDA levels were reversed in mice with LA early intervention (*p* < 0.05). Although LA treatment alone has no significant change (*p* > 0.05), a decreasing trend can be observed in the iNOS levels in the serum. Meanwhile, after *S*. Typhimurium infection, an increasing trend can be observed compared to CK group, even though there was no significant difference between the two groups (*p* > 0.05). As shown in Figures 3B,C, after *S*. Typhimurium infection, the mRNA expression levels of *SOD1*, *SOD2* and *GSH-Px1* were significantly decreased in the liver (*p* < 0.05), while only *SOD2* and *GSH-Px1* mRNA expression levels were significantly decreased in the duodenum (*p* < 0.05). LA treatment alone significantly elevated the mRNA expression levels of *SOD1*, *SOD2* and *HO-1* in the liver (*p* < 0.05), as well as *SOD2* in the duodenum (*p* < 0.05). Compared with the ST group, the mRNA expression levels of *SOD1*, *SOD2* in the liver were significantly higher in the LA + ST group (*p* < 0.05), while in the duodenum, *SOD2* mRNA expression was significantly higher (*p* < 0.05), and *GSH-Px1* mRNA expression level were also increased, but the difference was not statistically significant (*p* > 0.05). These findings indicated that early intervention of LA can help to alleviate the oxidative response caused by *S*. Typhimurium.

### 3.4 Effect of LA on the p62-Keap1-Nrf2 signaling pathway in *Salmonella* Typhimurium-induced mice

**Figure 4:**
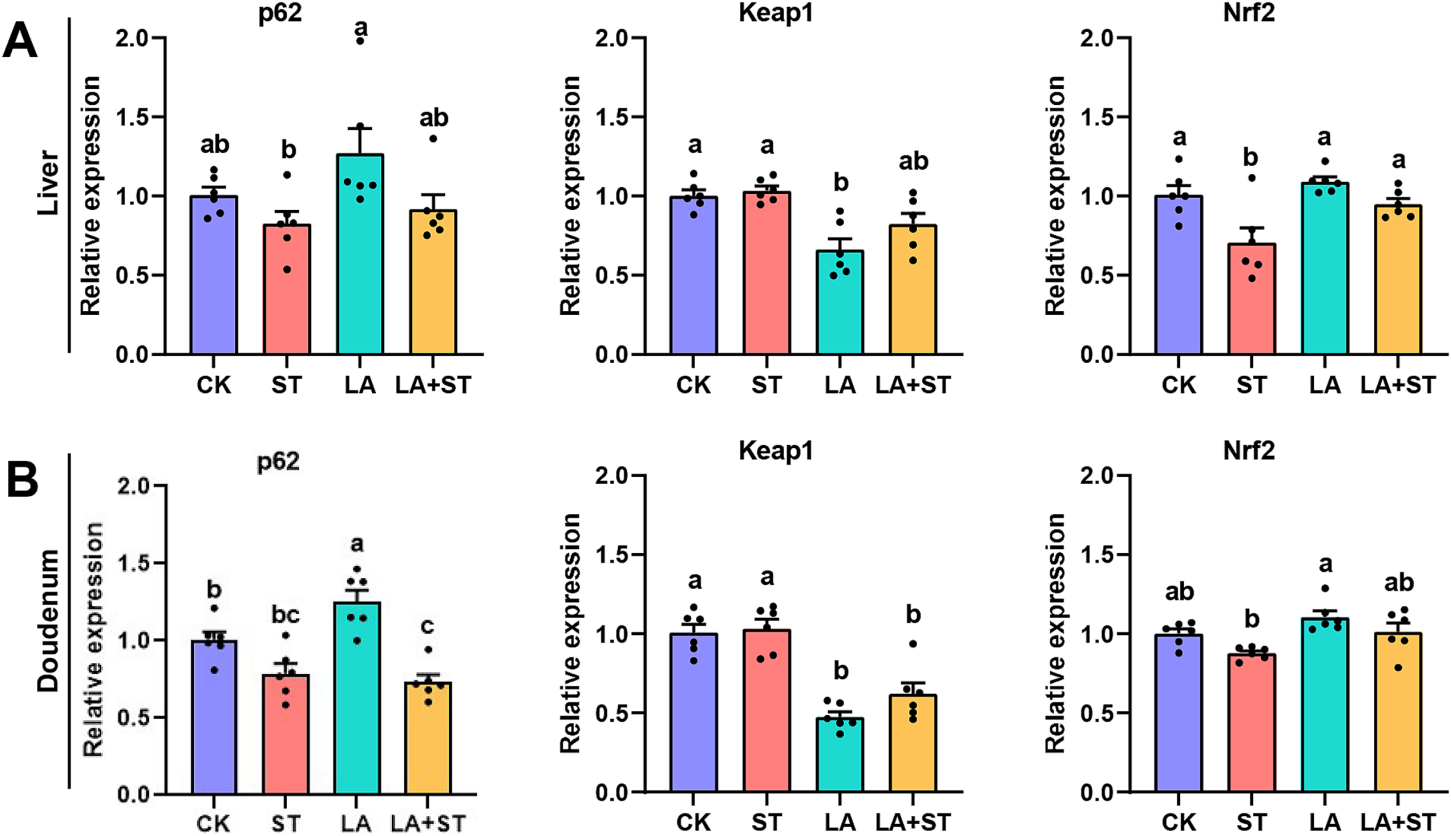
Effect of LA on the p62-Keap1-Nrf2 signaling pathway in *S*. Typhimurium-induced mice. The mRNA expression levels of *p62*, *Keap1*, and *Nrf2* in the liver **(A)** and duodenum **(B)** were detected using qPCR (6 mice per group). Data are expressed as mean ± SEM. Different lowercase letters on columns indicate a significant difference at the 0.05 level.

In order to study the changes of p62-Keap1-Nrf2 pathway during LA alleviating *S*. Typhimurium infected mice, we used qPCR to detect the mRNA expression levels of *p62*, *Keap1*, and *Nrf2* in this pathway. As shown in Figure 4, compared with those in the CK group, the mRNA expression levels of *Nrf2* in the liver was significantly reduced after *S*. Typhimurium infection (*p* < 0.05), while LA treatment alone increased the mRNA expression levels of duodenal *p62* (*p* < 0.05), and decreased *Keap1* mRNA expression in the liver and duodenum (*p* < 0.05). Compared with the ST group, *Nrf2* mRNA expression was significantly higher in the liver (*p* < 0.05) and *Keap1* mRNA expression was significantly lower (*p* < 0.05) in the duodenum of mice of the LA + ST group. Based on these results, we hypothesized that LA exerts anti-inflammatory/oxidative responses that are closely related to the p62-Keap1-Nrf2 signaling pathway.

### 3.5 Effect of LA on cecal microbiota in *Salmonella* Typhimurium-induced mice

**Figure 5:**
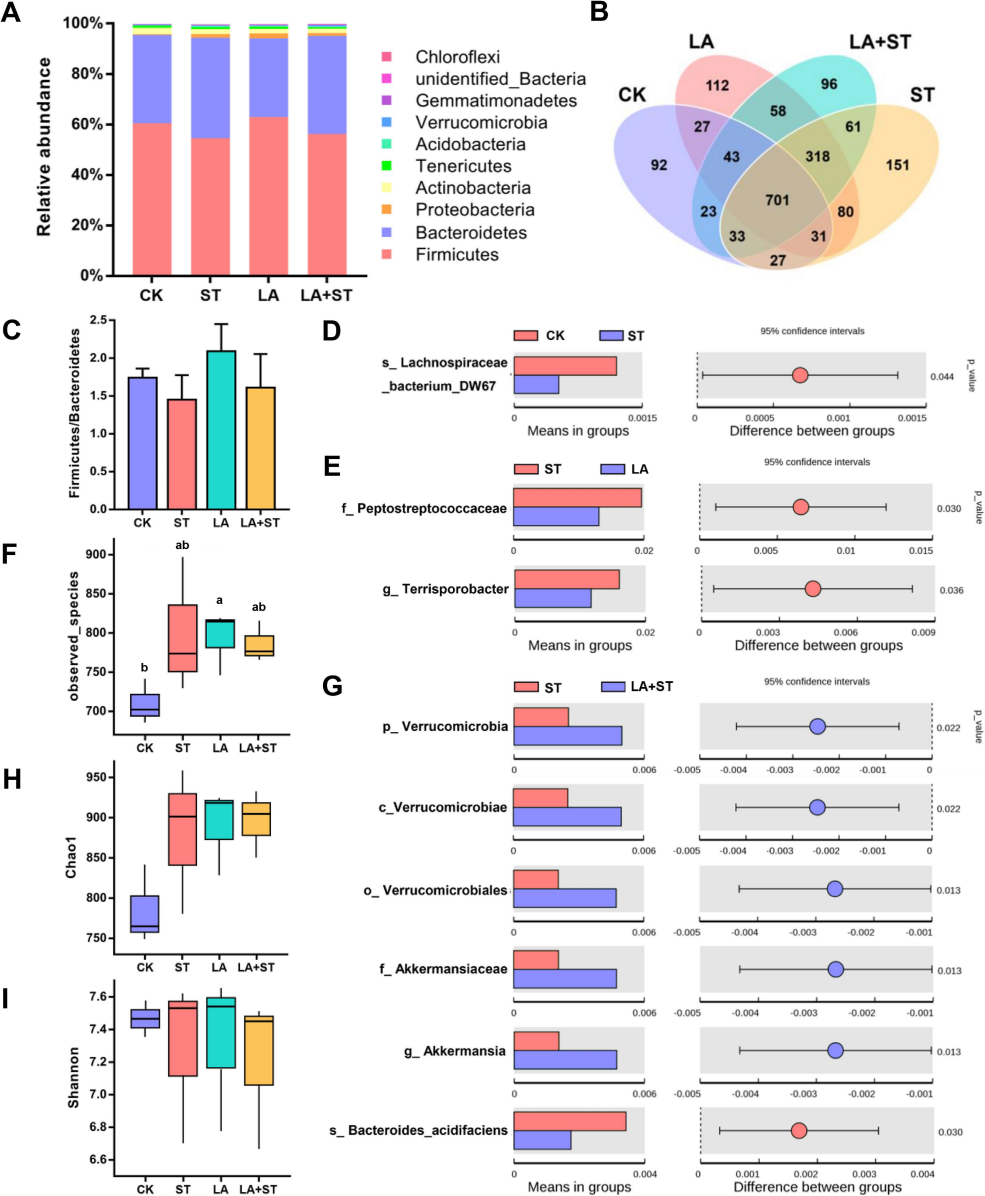
Effect of LA on cecal microbiota in *S*. Typhimurium-induced mice. **(A)** Relative abundances of the top 10 phylum species. **(B)** Unique and shared cecal OTUs among different groups shown in a Venn diagram. **(C)** Ratio of Firmicutes/Bacteroidetes histogram. **(D)** Student’s *t*-test of species differences between CK and ST groups with *p*-value corrected for multiple comparisons using the false discovery rate (FDR) method. **(E)** Student’s *t*-test of species differences between ST and LA groups with *p*-values corrected for multiple comparisons using the FDR method. **(F)** Number of bacterial species among different groups estimated by the observed species value. **(G)** Student’s *t*-test of species differences between ST and LA + ST groups with *p*-values corrected for multiple comparisons using the FDR method. **(H)** Bacterial richness among different groups estimated by the Chao1 index. **(I)** Bacterial diversity among different groups estimated by the Shannon index (3 mice per group). Data are presented as mean ± SEM. *p < 0.05.

To investigate the relationship between LA in alleviating the inflammatory/oxidative responses and intestinal microbiota in *S*. Typhimurium-infected mice, we analyzed the structural changes of the cecum microbial community using 16S rDNA amplicon sequencing (Figure 5). After splicing, quality control, and filtering chimeras on the raw data obtained from Il-lumina NovaSeq sequencing, 609,223 effective tags comprising 251,153,093 bp were obtained. Firmicutes and Bacteroidetes were the major phyla in the mouse cecum, and they collectively accounted for about 95% of each treatment group (Figure 5A). The proportions of Firmicutes and Bacteroidetes were higher in the LA + ST group than in the ST group, but the difference was not statistically significant (*p* > 0.05) (Figure 5C). Based on alpha diversity analysis, the highest number of observed species occurred in the LA group, and there was a significant difference with the CK group (*p* > 0.05) (Figure 5F). The Chao1 index was the lowest in the CK group, but was not significantly different from those of the other three groups (*p* > 0.05) (Figure 5H). The Shannon index was the highest in the LA group but was not significantly different from those of the other groups (*p* > 0.05) (Figure 5I). In addition, Figure 5B shows that the numbers of unique OTUs in the CK, ST, LA, and LA + ST groups were 92, 151, 112, and 96 respectively, indicating that LA and *S*. Typhimurium could enhance the microbial community richness and diversity of the mouse cecum to some extent.

In order to further understand the variation in the microbial community between treatment groups. We used Student’s *t*-test to screen for species with significant differences between groups at the phylum to species levels. When compared with the CK group, *Lachnospirace-ae_bacterium_DW67* was lower in abundance in the cecum of *S*. Typhimurium-infected mice (Figure 5D). The abundances of *Peptostreptococcaceae* and *Terrisporobacter* were much lower in the LA group than in the ST group (Figure 5E). Moreover, we compared the microbial composition between LA + ST and ST groups and found that *Akkermansia* were significantly enriched in the LA + ST group, while *Bacteroides acidifaciens* was significantly enriched in the ST group (Figure 5G).

### 3.6 Effect of LA on short chain fatty acids in *Salmonella* Typhimurium-induced mice

**Figure 6:**
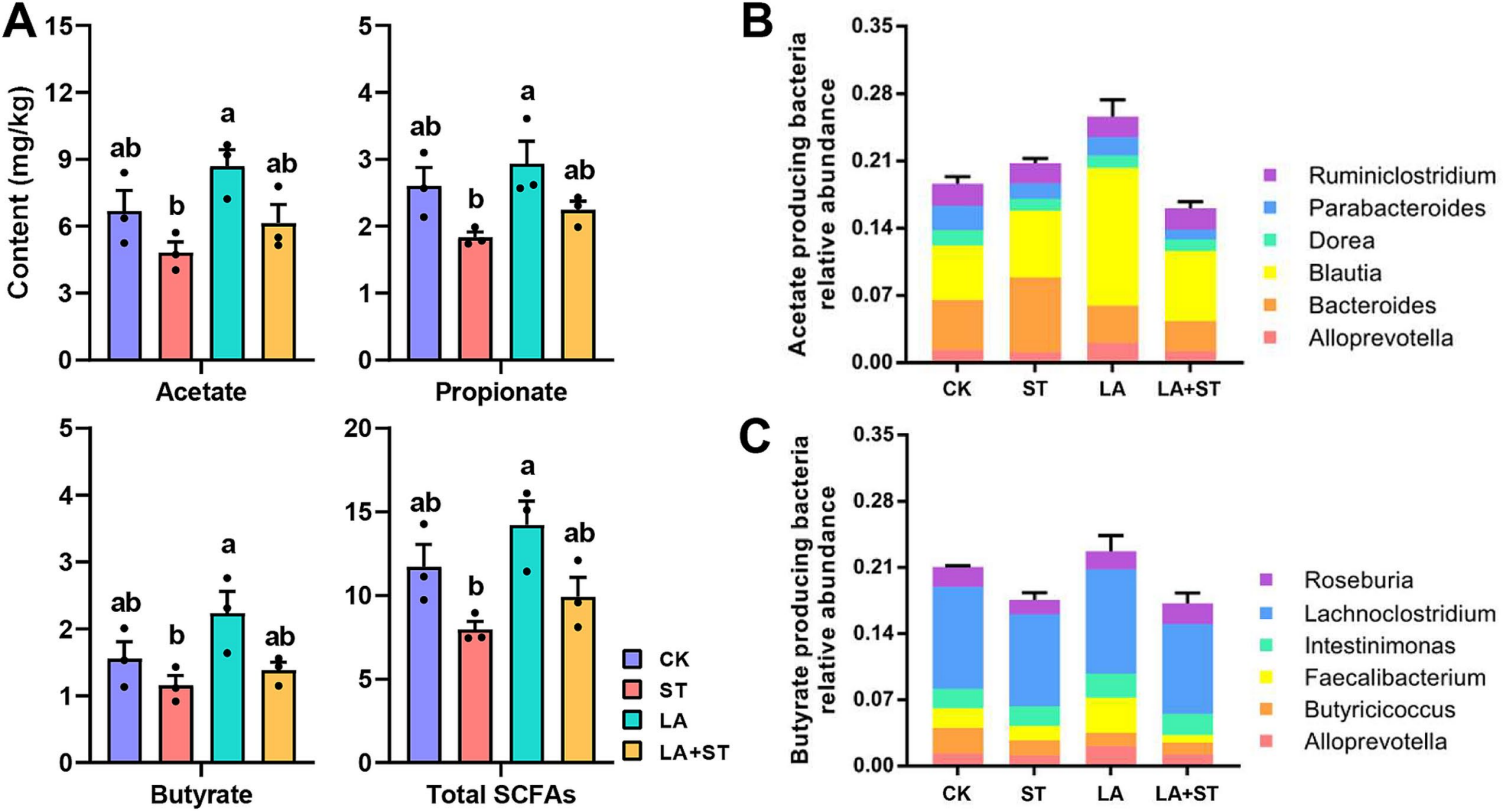
Effects of LA and *S*. Typhimurium on cecal short-chain fatty acid contents, acetate-producing bacteria and butyrate-producing bacteria relative abundance. **(A)** The contents of acetate, propionate, butyrate, and total short-chain fatty acids in the cecum were analyzed. **(B)** Acetate-producing bacteria relative abundance. **(C)** Butyrate-producing bacteria relative abundance. (3 mice per group) Data are mean ± SEM. Significant differences of short-chain fatty acid contents in four different groups were analyzed using one-way analysis of variance with Duncan’s method. Different lowercase letters on columns indicate a significant difference at the 0.05 level.

Figure 6A shows that the concentrations of acetate, propionate, butyrate, and total SCFAs in the cecum contents of mice in the ST group were significantly lower than those in the LA group (*p* < 0.05). Acetate, propionate, butyrate, and total SCFA concentrations were higher in the LA + ST group than in the ST group, but there was no statistically significant difference (*p* > 0.05). This suggests that LA early intervention can reverse the *S*. Typhimurium-induced short-chain fatty acid decrease to some extent. To gain more insight into the effects of LA and *S*. Typhimurium on acetate- and butyrate-producing bacteria in the cecum, we analyzed the relative abundances of butyrate- and acetate-producing bacteria in each group (Figures 6B,C) (Xia et al., 2021). These results showed that LA has the ability to promote the increase of butyrate- or acetate-producing bacterial abundance.

### 3.7 Spearman correlation between cecal microbiota, SCFAs and serum indicators

**Figure 7:**
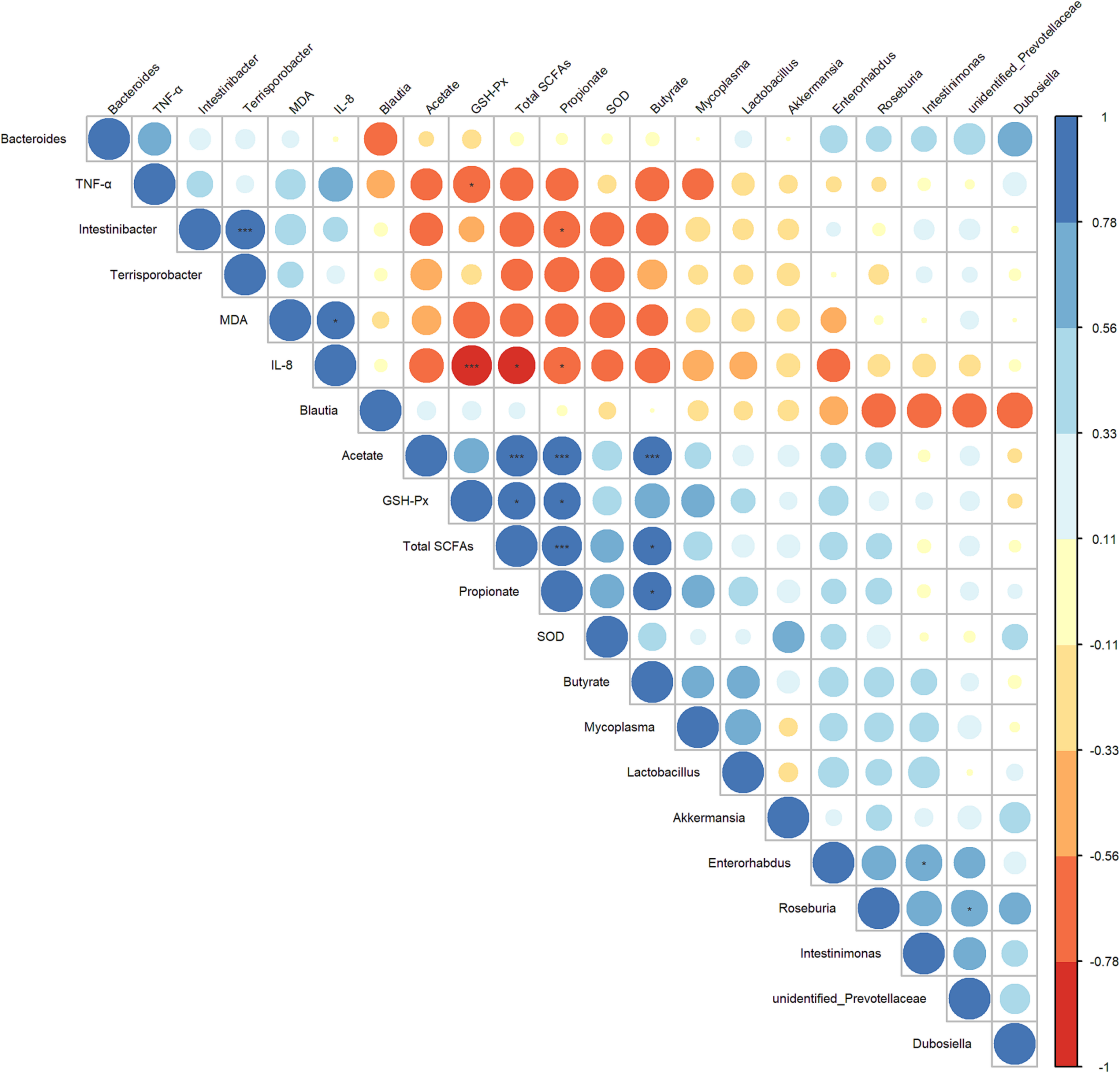
Heatmap of the correlation analysis conducted among bacterial genera, cecal SCFAs, and serum oxidative/inflammatory response indicators. Spearman’s correlation coefficient was used in the analysis. The correlation coefficient plot is represented by circles, with the size and color intensity of the circles corresponding to the magnitude of the correlation coefficient. Blue indicates a positive correlation, while red signifies a negative correlation. Larger circles denote higher absolute values of the correlation coefficient. *p* values were adjusted for multiple comparisons to control the false discovery rate with the use of the Benjamini–Hochberg method. *p ≤ 0.1, **p ≤ 0.05, and ***p ≤ 0.01.

Next, to further explore the interrelationship between inflammatory/oxidative response markers, cecal microbiota, and their metabolites, we constructed a Spearman correlation heatmap. As shown in Figure 7, the concentrations of propionate and total SCFAs showed a significant negative correlation with IL-8 level (*r* = 0.782, *p* = 0.0494; *r* = 0.797, *p* = 0.0382) and a significant positive correlated with the GSH-Px level (*r* = 0.797, *p* = 0.0382; *r* = 0.782, *p* = 0.0494). The abundance of *Intestinibacter* was significantly negatively correlated with the concentrations of propionate (*r* = −0.758, *p* = 0.0494), and was positively correlated with the abundance of *Terrisporobacter* (*r* = 0.895, *p* ≤ 0.001). The level of IL-8 and TNF-α exhibited a significant negative correlation with the GSH-Px (*r* = −0.937, *p* ≤ 0.001; *r* = −0.769, *p* = 0.0485). Based on these results, we hypothesized that the anti-inflammatory/antioxidant responses exerted by LA are closely related to the beneficial regulation of cecal microbiota and SCFA production.

## 4 Discussion

*S*. Typhimurium could evade the host’s immune defense system and invade the intestinal epithelium, disrupting the tight junction structure and function and in turn causing disruption of the intestinal mucosal barrier, ultimately triggering gastrointestinal diseases (Fàbrega and Vila, 2013; Zhang et al., 2019). *S*. Typhimurium use phagocytes (e.g., macrophages) to enter the lymphatic system and spread to systemic tissues (including liver, spleen, and lymph nodes), consequently establishing and maintaining a chronic infection in the host (Behnsen et al., 2015; Gal-Mor, 2018). The liver plays an important role in metabolic processes, participating in a variety of physiological activities such as detoxification, immune response, and nutrient trans-formation (Trefts et al., 2017). Accordingly, liver and intestinal damage are important markers of host response to *S*. Typhimurium infection. In the preliminary stages of the experiment, to investigate the distribution of *S*. Typhimurium in mouse organs, we measured the bacterial burden of *S*. Typhimurium in the spleen, liver, and cecum of mice 24 h post-infection. No *S*. Typhimurium was detected in the spleen or liver of the CK, ST, LA, and LA + ST groups. The number of *S*. Typhimurium in the cecum of the CK, ST, LA, and LA + ST groups were 0, 3.15, 0, and 1.58 lg (CFU/g), respectively. These results suggest that LA may reduce the susceptibility of mice to *S*. Typhimurium, decrease *S*. Typhimurium colonization, and lower the intestinal pathogenic bacterial load. In this study, we found that after *S*. Typhimurium infection, mice showed lymphocyte aggregation and punctate necrosis in the liver, and the normal structures of the duodenal villi and crypt were largely lost. The mice exhibited lymphocyte infiltration and severe ulceration of the lamina propria, which could be alleviated by early intervention with LA. The relatively mild inflammatory infiltration in the LA + ST group may be attributed to the protective effects of LA, which were administered continuously prior to the inflammatory stimulus. When hepatocytes are exposed to inflammation, such as in the case of *S*. Typhimurium infection, increased permeability of the liver sinusoidal wall may lead to sinusoidal dilation. Compared to the CK group, the overall liver changes in the LA group were minimal, and the slight infiltration observed may be due to individual mouse variability. The histological score also further confirmed the positive effect of early intervention with LA. In addition, the role of tight junctions between each cell determines the integrity of the tissue barrier. Pathogens can disrupt the structure and function of tight junctions by changing tight junction proteins (ZO-1, occludin, and claudin), resulting in increased tissue permeability (Zhang et al., 2019). Wang et al. (2018) found that *Lactobacillus plantarum* can alleviate the decline of *ZO-1* expression in the cecum of newborn laying hens caused by *S*. Typhimurium infection. Splichalova et al. (2019) found that *S*. Typhimurium can reduce the expression of *occludin* mRNA and increase the expression of *claudin-1* mRNA in ileum and colon of piglets; *Lactobacillus rhamnosus* GG alone increased the expression of *occludin* mRNA in ileum and colon. In our study, we found that *S*. Typhimurium infection reduced the expression of *ZO-1* in the liver, as well as *ZO-1*, *occludin*, and *claudin-1* in the duodenum at the transcriptional level. *Lactobacillus* can promote the expression of tight junction protein in different animal models. These results provide some hints for us in the follow-up study of the effect of LA on intestinal barrier function.

Oxidative stress and inflammation are closely related in resisting pathogen invasion and excessive inflammation or oxidative stress will endanger the health of the host (Chakraborty et al., 2020; Chainy and Sahoo, 2020; Deramaudt et al., 2013). The increase of proinflammatory cytokine TNF-α will increase or amplify the inflammatory cascade and cause tissue damage of the host. *S.* Typhimurium LT2 caused an increase in IL-8 levels in serum and jejunum of piglets, which was reduced by the intervention of *Lactobacillus rhamnosus* GG (Splichalova et al., 2019). In our early study, LA caused TNF-α and IFN-c (interferon-c) concentration decreased, and protected piglets from LPS induced inflammatory response (Qiao et al., 2015). In this experiment, LA alleviated the increase of serum TNF and IL-8 in mice caused by *S*. Typhimurium. Therefore, we hypothesized that LA may help to protect mice from damage caused by *S*. Typhimurium infection by attenuating the inflammatory response and oxidative stress.

Some studies have demonstrated that *Lactobacillus* increases the antioxidant capacity of animal intestines and liver. *Lactobacillus plantarum* alleviates weaning stress in goats by reducing intestinal MDA and increasing SOD and GSH-PX enzyme activities (Chen et al., 2020). And research has found that *Lactobacillus paracasei ssp. paracasei YBJ01* has a promoting effect on the protein expression of SOD1 and SOD2 in the mice liver (Suo et al., 2018) In a high-fat-fed rat model of non-alcoholic fatty liver disease, Chen et al. (2018) found that *Lactobacillus mali* APS1 intervention significantly reduced MDA levels and promoted SOD activity in the liver, improving antioxidant capacity. The results of this study also demonstrated that LA had a certain resistance to the oxidation caused by *S*. Typhimurium after its early intervention. Therefore, we hypothesized that LA may help to protect mice from damage caused by *S*. Typhimurium infection by attenuating the inflammatory response and oxidative stress.

Hos et al. (2017) demonstrated that *S*. Typhimurium exploits can upregulate the mitochondrial phosphatase Pgam5 of macrophages, and Pgam5 interacts with Nrf2 to inhibit the transcription and expression of Nrf2 dependent antioxidant genes. With the weakening of cell antioxidant capacity, mitochondria are more prone to oxidative damage, leading to cell energy consumption, inducing p62 autophagy degradation, affecting the interaction between p62 and Keap1, which led to the decline of host antioxidant capacity. In addition, the regulation of animals is a dynamic balance, and the relevant indicators may be affected by the infected object, infection duration and so on. When *S*. Typhimurium infected bone marrow macrophages, the protein expression of p62 and Nrf2 decreased and Keap1 increased 2 h after infection, but the protein expression of p62, Keap1 and Nrf2 returned to a level similar to the normal value 6 h after infection (Hos et al., 2017). In the research on probiotics, it was found that probiotics can promote the expression of *Nrf2* in animal tissues. Xu et al. (2019) demonstrated that the expression of Nrf2 protein in the heart of obese mice exposed to intermittent hypoxia would be reduced, and oral administration of *Lactobacillus rhamnosus* GG would help it return to normal levels. Similarly, our study found that *Nrf2* mRNA expression level in the liver was significantly reduced and *Keap1* mRNA expression level in the duodenum was significantly increased in the mice infected with *S*. Typhimurium, while the mRNA expression level of *Nrf2* and *Keap1* were reversed in mice with LA early intervention. This suggests that the role of LA in alleviating inflammation and oxidative responses in infected *S*. Typhimurium mice might be associated with the activation of the p62-Keap1-Nrf2 signaling pathway, a defense mechanism against inflammation and oxidative stress.

A growing number of studies suggest that changes in microbial communities may be closely related to the degree of inflammation and oxidative response (Tang et al., 2017; Brugiroux et al., 2016; Luca et al., 2019). SCFAs are important metabolites for the microorganisms to maintain homeostasis in the intestine (Parada Venegas et al., 2019). In the present study, the content of SCFAs in the LA group increased compared with the CK group, and alleviated the decrease of SCFAs caused by *S*. Typhimurium, and the relative abundance of acetate producing bacteria and butyate producing bacteria in LA group was higher than that in other groups. Butyrate can inhibit the activation of NF-κB and improve the intestinal barrier (Segain et al., 2000). The depletion of butyrate-producing microbes in the intestine reduces intracellular butyrate sensor peroxisome proliferator-activated receptor *γ* (PPAR-γ) signal transduction, increasing iNOS and nitrate levels, is conducive to the expansion of pathogenic *Escherichia* and *Salmonella*. Propionate can also inhibit the colonization of *Salmonella* (Jacobson et al., 2018). This study demonstrated that *Terrisporobacter* is significantly enriched in the ST group compared with the LA group. Cai et al. (2019) found that infants with a high degree of oxidative stress have a high relative abundance of *Terrisporobacter*. In children with type I diabetes, *Terrisporobacter* is negatively correlated with anti-inflammatory factor IL-10, and the intake of probiotics reduces the abundance of *Terrisporobacter*. *Intestinibacter* is significantly elevated in patients with chronic enteritis Crohn’s disease (Forbes et al., 2018). In this study, *Intestinibacter* was significantly positively correlated with *Terrisporobacter*, and significantly negatively correlated with propionate. These results may suggest that a series of host changes caused by the intake of *S*. Typhimurium may be related to the relative abundance of *Terrisporobacter* and *Intestinibacter*. SCFA propionate have the ability to decrease pro-inflammatory cytokine expression to potentiate the generation of *de novo* Treg cell (Arpaia et al., 2013). Cheng et al. (2019) found that propionate treatment in advance can increase the activities of the antioxidant enzymes, including CAT, SOD and GSH-Px, in mitochondria. Our correlation analysis showed that the concentrations of propionate showed a significant negative correlation with IL-8 level and a significant positive correlation with GSH-Px, is consistent with the above findings. Based on previous studies and our findings, *S*. Typhimurium infection is conducive to the increase of inflammation promoting microorganisms in the intestine, such as *Bacteroides acidifaciens*, *Intestinibacter*, and *Terrisporobacter*, and the reduction of short chain fatty acid producing bacteria and their products. The addition of LA reduced *Bacteroides* species in the gut and promoted the growth of *Akkermansia* and SCFA producing bacteria, which played a protective role in the gut.

**Figure 8:**
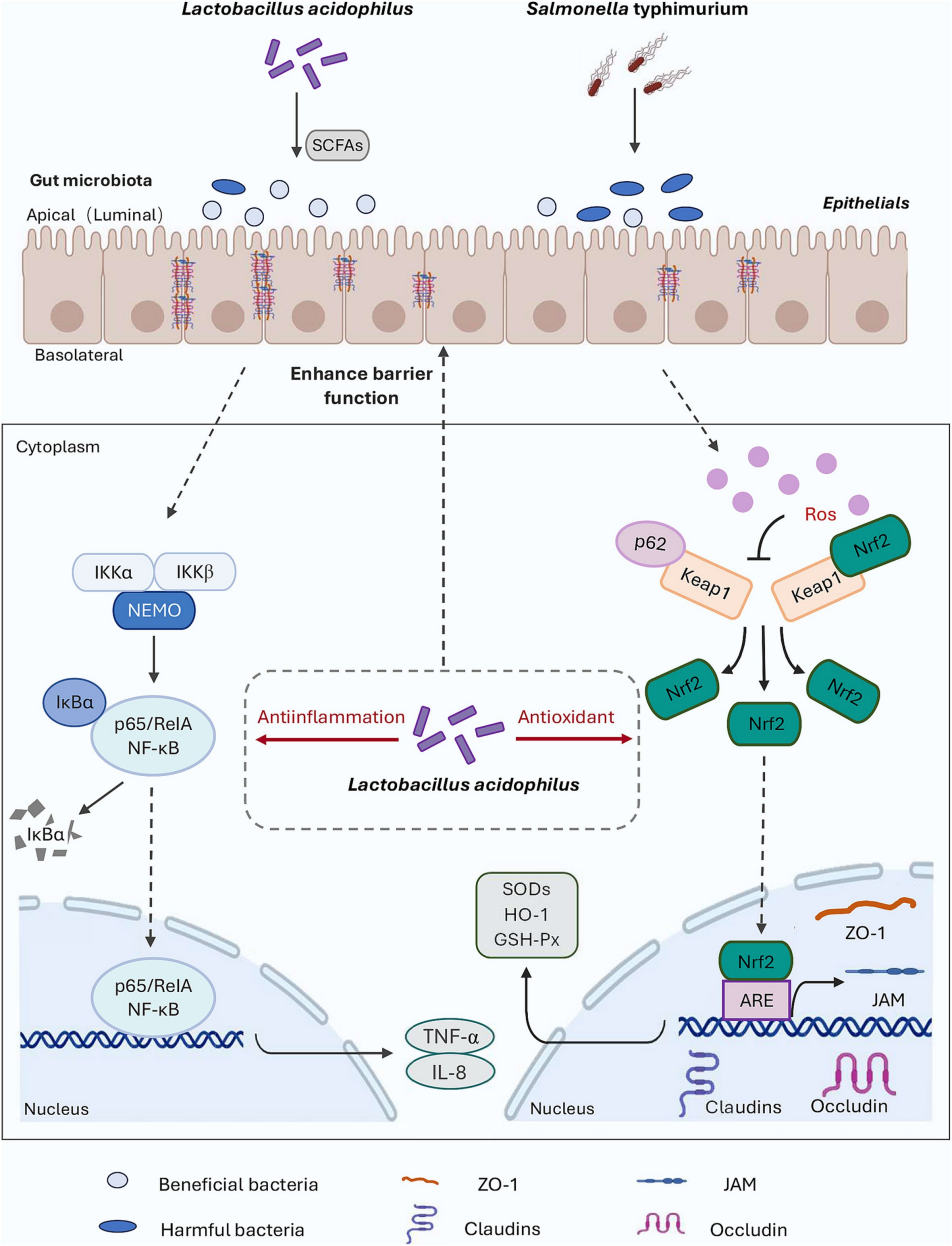
Diagram outlining the mechanism of LA on alleviate *S*. Typhimurium-induced inflammation and oxidative responses.

In this study, *S*. Typhimurium was used as the disease-causing model for *in vivo* testing. The results indicated that the alleviation of inflammation and oxidative damage by LA was closely linked to the p62-Keap1-Nrf2 signaling pathway and the structure of the intestinal microbial community, as illustrated by the potential mechanism of action in Figure 8. Upon *S*. Typhimurium infection of the gut, the normal intestinal microbiota is altered, leading to an increase in harmful substances, enhanced gut barrier permeability, and stimulation of cells to produce high levels of ROS and pro-inflammatory cytokines (such as TNF-α and IL-8), which cause damage to intestinal epithelial cells. When LA enters the gut, it can upregulate the expression of genes associated with the p62-Keap1-Nrf2 signaling pathway (e.g., SOD, GSH-Px), as well as increase the mRNA expression of tight junction proteins. This response helps to alleviate the inflammation and oxidative damage induced by *S*. Typhimurium. Additionally, LA can modulate the gut microbiota by competing for colonization sites, thereby reducing the proliferation of harmful bacteria and promoting the production of SCFAs. This supports the maintenance of normal gut function and stability and helps mitigate the inflammation and oxidative damage caused by ST infection. However, this study did not involve the knockout of the Keap1 gene to further verify the role of the p62-Keap1-Nrf2 signaling pathway in the anti-inflammatory and antioxidant effects of LA, which remains an area for future investigation.

## 5 Conclusion

Taken together, the results indicate that LA early intervention may alleviate *S*. Typhimurium-induced inflammation and oxidative responses in mice by activating the p62-Keap1-Nrf2 signaling path-way and regulating the gut microbial community to some extent.

## Data Availability

The datasets presented in this study can be found in online repositories. The names of the repository/repositories and accession number(s) can be found in the article/Supplementary material.

## References

[ref2] Arpaia N. Campbell C. Fan X. Dikiy S. van der Veeken J. deRoos P.. (2013). Metabolites produced by commensal bacteria promote peripheral regulatory T-cell generation. Nature 504, 451–455. doi: 10.1038/nature12726, PMID: 24226773 PMC3869884

[ref3] Ashrafi G. Schwarz T. L. (2013). The pathways of mitophagy for quality control and clearance of mitochondria. Cell Death Differ. 20, 31–42. doi: 10.1038/cdd.2012.81, PMID: 22743996 PMC3524633

[ref4] Azcarate-Peril M. A. Altermann E. Hoover-Fitzula R. L. Cano R. J. Klaenhammer T. R. (2004). Identification and inactivation of genetic loci involved with *Lactobacillus acidophilus* acid tolerance. Appl. Environ. Microb. 70, 5315–5322. doi: 10.1128/AEM.70.9.5315-5322.2004, PMID: 15345415 PMC520879

[ref5] Behnsen J. Perez-Lopez A. Nuccio S. P. Raffatellu M. (2015). Exploiting host immunity: the *Salmonella* paradigm. Trends Immunol. 36, 112–120. doi: 10.1016/j.it.2014.12.003, PMID: 25582038 PMC4323876

[ref7] Bindu S. Pal C. Dey S. Goyal M. Alam A. Iqbal M. S.. (2011). Translocation of heme oxygenase-1 to mitochondria is a novel cytoprotective mechanism against non-steroidal anti-inflammatory drug-induced mitochondrial oxidative stress, apoptosis, and gastric mucosal injury. J. Biol. Chem. 286, 39387–39402. doi: 10.1074/jbc.M111.279893, PMID: 21908612 PMC3234763

[ref8] Blake D. R. Allen R. E. Lunec J. (1987). Free radicals in biological systems--a review orientated to inflammatory processes. Brit. Med. Bull. 43, 371–385. doi: 10.1093/oxfordjournals.bmb.a072188, PMID: 3319034

[ref9] Brugiroux S. Beutler M. Pfann C. Garzetti D. Ruscheweyh H. J. Ring D.. (2016). Genome-guided design of a defined mouse microbiota that confers colonization resistance against *Salmonella enterica* serovar typhimurium. Nat. Microbiol. 2:16215. doi: 10.1038/nmicrobiol.2016.215, PMID: 27869789

[ref10] Cai C. Zhang Z. Morales M. Wang Y. Khafipour E. Friel J. (2019). Feeding practice influences gut microbiome composition in very low birth weight preterm infants and the association with oxidative stress: a prospective cohort study. Free Radic. Biol. Med. 142, 146–154. doi: 10.1016/j.freeradbiomed.2019.02.032, PMID: 30851363

[ref11] Caporaso J. G. Kuczynski J. Stombaugh J. Bittinger K. Bushman F. D. Costello E. K.. (2010). QIIME allows analysis of high-throughput community sequencing data. Nat. Methods 7, 335–336. doi: 10.1038/nmeth.f.303, PMID: 20383131 PMC3156573

[ref12] Chainy G. Sahoo D. K. (2020). Hormones and oxidative stress: an overview. Free Radic. Res. 54, 1–26. doi: 10.1080/10715762.2019.1702656, PMID: 31868060

[ref13] Chakraborty S. Liu L. Fitzsimmons L. Porwollik S. Kim J. S. Desai P.. (2020). Glycolytic reprograming in *Salmonella* counters NOX2-mediated dissipation of ΔpH. Nat. Commun. 11:1783. doi: 10.1038/s41467-020-15604-2, PMID: 32286292 PMC7156505

[ref14] Chen Y. T. Lin Y. C. Lin J. S. Yang N. S. Chen M. J. (2018). Sugary kefir strain *Lactobacillus mali* APS1 ameliorated hepatic steatosis by regulation of SIRT-1/Nrf-2 and gut microbiota in rats. Mol. Nutr. Food Res. 62:e1700903. doi: 10.1002/mnfr.201700903, PMID: 29508520

[ref15] Chen K. Liu Y. Cheng Y. Yan Q. Zhou C. He Z.. (2020). Supplementation of *Lactobacillus plantarum* or *Macleaya cordata* extract alleviates oxidative damage induced by weaning in the lower gut of Young goats. Animals 10:548. doi: 10.3390/ani10040548, PMID: 32218197 PMC7222368

[ref16] Cheng X. Xi Z. Liang H. Sun Y. Zhong Z. Wang B.. (2019). Melatonin prevents mice cortical astrocytes from hemin-induced toxicity through activating PKCα/Nrf2/HO-1 signaling in vitro. Front. Neurosci. 13:760. doi: 10.3389/fnins.2019.00760, PMID: 31404262 PMC6669962

[ref17] Cheng Y. Mai Q. Zeng X. Wang H. Xiao Y. Tang L.. (2019). Propionate relieves pentylenetetrazol-induced seizures, consequent mitochondrial disruption, neuron necrosis and neurological deficits in mice. Biochem. Pharmacol. 169:113607. doi: 10.1016/j.bcp.2019.08.009, PMID: 31491413

[ref18] Chiu C. J. McArdle A. H. Brown R. Scott H. J. Gurd F. N. (1970). Intestinal mucosal lesion in low-flow states. I. A morphological, hemodynamic, and metabolic reappraisal. Arch. Surg. 101, 478–483. doi: 10.1001/archsurg.1970.01340280030009, PMID: 5457245

[ref19] Deramaudt T. B. Dill C. Bonay M. (2013). Regulation of oxidative stress by Nrf2 in the pathophysiology of infectious diseases. Med. Mal. Infect. 43, 100–107. doi: 10.1016/j.medmal.2013.02.004, PMID: 23499316

[ref20] Ding L. A. Li J. S. Li Y. S. Zhu N. T. Liu F. N. Tan L. (2004). Intestinal barrier damage caused by trauma and lipopolysaccharide. World J. Gastroenterol. 10, 2373–2378. doi: 10.3748/wjg.v10.i16.2373, PMID: 15285022 PMC4576291

[ref21] Ducarmon Q. R. Zwittink R. D. Hornung B. V. H. van Schaik W. Young V. B. Kuijper E. J. (2019). Gut microbiota and colonization resistance against bacterial enteric infection. Microbiol. Mol. Biol. Rev. 83, e00007–e00019. doi: 10.1128/MMBR.00007-19, PMID: 31167904 PMC6710460

[ref22] Eaves-Pyles T. Murthy K. Liaudet L. Virág L. Ross G. Soriano F. G.. (2001). Flagellin, a novel mediator of *Salmonella*-induced epithelial activation and systemic inflammation: I kappa B alpha degradation, induction of nitric oxide synthase, induction of proinflammatory mediators, and cardiovascular dysfunction. J. Immunol. 166, 1248–1260. doi: 10.4049/jimmunol.166.2.1248, PMID: 11145708

[ref23] Fàbrega A. Vila J. (2013). *Salmonella enterica* serovar Typhimurium skills to succeed in the host: virulence and regulation. Clin. Microbiol. Rev. 26, 308–341. doi: 10.1128/CMR.00066-12, PMID: 23554419 PMC3623383

[ref24] Forbes J. D. Chen C. Y. Knox N. C. Marrie R. A. El-Gabalawy H. de Kievit T.. (2018). A comparative study of the gut microbiota in immune-mediated inflammatory diseases-does a common dysbiosis exist? Microbiome 6:221. doi: 10.1186/s40168-018-0603-4, PMID: 30545401 PMC6292067

[ref25] Gal-Mor O. (2018). Persistent infection and Long-term carriage of Typhoidal and Nontyphoidal *salmonellae*. Clin. Microbiol. Rev. 32, e00088–e00018. doi: 10.1128/CMR.00088-18, PMID: 30487167 PMC6302356

[ref26] Gewirtz A. T. Simon P. O. Jr. Schmitt C. K. Taylor L. J. Hagedorn C. H. O'Brien A. D.. (2001). *Salmonella* Typhimurium translocates flagellin across intestinal epithelia, inducing a proinflammatory response. J. Clin. Invest. 107, 99–109. doi: 10.1172/JCI10501, PMID: 11134185 PMC198545

[ref27] Goh Y. J. Klaenhammer T. R. (2014). Insights into glycogen metabolism in *Lactobacillus acidophilus*: impact on carbohydrate metabolism, stress tolerance and gut retention. Microb. Cell Factories 13:94. doi: 10.1186/s12934-014-0094-3, PMID: 25410006 PMC4243779

[ref28] Haas B. J. Gevers D. Earl A. M. Feldgarden M. Ward D. V. Giannoukos G.. (2011). Chimeric 16S rRNA sequence formation and detection in sanger and 454–pyrosequenced PCR amplicons. Genome Res. 21, 494–504. doi: 10.1101/gr.112730.110, PMID: 21212162 PMC3044863

[ref29] Hernandez L. D. Pypaert M. Flavell R. A. Galán J. E. (2003). A *Salmonella* protein causes macrophage cell death by inducing autophagy. J. Cell Biol. 163, 1123–1131. doi: 10.1083/jcb.200309161, PMID: 14662750 PMC2173598

[ref30] Herrero-Fresno A. Olsen J. E. (2018). *Salmonella* Typhimurium metabolism affects virulence in the host-a mini-review. Food Microbiol. 71, 98–110. doi: 10.1016/j.fm.2017.04.016, PMID: 29366476

[ref31] Ho E. Karimi Galougahi K. Liu C. C. Bhindi R. Figtree G. A. (2013). Biological markers of oxidative stress: applications to cardiovascular research and practice. Redox Biol. 1, 483–491. doi: 10.1016/j.redox.2013.07.006, PMID: 24251116 PMC3830063

[ref32] Hos N. J. Ganesan R. Gutiérrez S. Hos D. Klimek J. Abdullah Z.. (2017). Type I interferon enhances necroptosis of *Salmonella* Typhimurium-infected macrophages by impairing antioxidative stress responses. J. Cell Biol. 216, 4107–4121. doi: 10.1083/jcb.201701107 29055012 PMC5716270

[ref33] Huang W. C. Pan C. H. Wei C. C. Huang H. Y. (2020). *Lactobacillus plantarum* PS128 improves physiological adaptation and performance in triathletes through gut microbiota modulation. Nutrients 12:2315. doi: 10.3390/nu12082315, PMID: 32752178 PMC7468698

[ref34] Ichimura Y. Waguri S. Sou Y. S. Kageyama S. Hasegawa J. Ishimura R.. (2013). Phosphorylation of p62 activates the Keap1–Nrf2 pathway during selective autophagy. Mol. Cell 51, 618–631. doi: 10.1016/j.molcel.2013.08.003, PMID: 24011591

[ref35] Jacobson A. Lam L. Rajendram M. Tamburini F. Honeycutt J. Pham T.. (2018). A gut commensal-produced metabolite mediates colonization resistance to Salmonella infection. Cell Host Microbe 24, 296–307.e7. doi: 10.1016/j.chom.2018.07.002, PMID: 30057174 PMC6223613

[ref36] Jiang X. Gu S. Liu D. Zhao L. Xia S. He X.. (2018). *Lactobacillus brevis* 23017 relieves mercury toxicity in the Colon by modulation of oxidative stress and inflammation through the interplay of MAPK and NF–κB signaling cascades. Front. Microbiol. 9:2425. doi: 10.3389/fmicb.2018.02425, PMID: 30369917 PMC6194351

[ref37] Kamel H. M. Hume S. P. Carr K. E. Marigold J. C. Michalowski A. (1988). Development of villous damage in mouse small intestine after local hyperthermia or irradiation. J. Submicrosc. Cytol. Pathol. 20, 185–193, PMID: 3370617

[ref38] Kang B. Kim C. Y. Hwang J. Jo K. Kim S. Suh H. J.. (2019). Punicalagin, a pomegranate–derived ellagitannin, suppresses obesity and obesity–induced inflammatory responses via the Nrf2/Keap1 signaling pathway. Mol. Nutr. Food Res. 63:e1900574. doi: 10.1002/mnfr.201900574 31444955

[ref39] Kayama H. Okumura R. Takeda K. (2020). Interaction between the microbiota, epithelia, and immune cells in the intestine. Annu. Rev. Immunol. 38, 23–48. doi: 10.1146/annurev-immunol-070119-115104, PMID: 32340570

[ref40] Keller M. D. Torres V. J. Cadwell K. (2020). Autophagy and microbial pathogenesis. Cell Death Differ. 27, 872–886. doi: 10.1038/s41418-019-0481-8, PMID: 31896796 PMC7205878

[ref41] Khaminets A. Behl C. Dikic I. (2016). Ubiquitin-dependent and independent signals in selective autophagy. Trends Cell Biol. 26, 6–16. doi: 10.1016/j.tcb.2015.08.010, PMID: 26437584

[ref42] Kim T. W. Shin J. S. Chung K. S. Lee Y. G. Baek N. I. Lee K. T. (2019). Anti-inflammatory mechanisms of Koreanaside a, a Lignan isolated from the flower of Forsythia koreana, against LPS-induced macrophage activation and DSS-induced colitis mice: the crucial role of AP-1, NF-κB, and JAK/STAT signaling. Cells 8:1163. doi: 10.3390/cells8101163, PMID: 31569788 PMC6829247

[ref43] Komatsu W. Kishi H. Yagasaki K. Ohhira S. (2018). Urolithin a attenuates pro–inflammatory mediator production by suppressing PI3–K/Akt/NF–κB and JNK/AP–1 signaling pathways in lipopolysaccharide–stimulated RAW264 macrophages: possible involvement of NADPH oxidase–derived reactive oxygen species. Eur. J. Pharmacol. 833, 411–424. doi: 10.1016/j.ejphar.2018.06.023, PMID: 29932926

[ref44] Lee T. S. Chau L. Y. (2002). Heme oxygenase–1 mediates the anti–inflammatory effect of interleukin–10 in mice. Nat. Med. 8, 240–246. doi: 10.1038/nm0302-240, PMID: 11875494

[ref45] Lépine A. F. P. de Wit N. Oosterink E. Wichers H. Mes J. de Vos P. (2018). *Lactobacillus acidophilus* attenuates Salmonella-induced stress of epithelial cells by modulating tight-junction genes and cytokine responses. Front. Microbiol. 9:1439. doi: 10.3389/fmicb.2018.01439, PMID: 30013538 PMC6036613

[ref46] Li C. Tan F. Yang J. Yang Y. Gou Y. Li S.. (2019). Antioxidant effects of Apocynum venetum tea extracts on d–galactose–induced aging model in mice. Antioxidants 8:381. doi: 10.3390/antiox8090381, PMID: 31500342 PMC6770887

[ref47] Li H. Zhang L. Chen L. Zhu Q. Wang W. Qiao J. (2016). *Lactobacillus acidophilus* alleviates the inflammatory response to enterotoxigenic *Escherichia coli* K88 via inhibition of the NF–κB and p38 mitogen–activated protein kinase signaling pathways in piglets. BMC Microbiol. 16:273. doi: 10.1186/s12866-016-0862-9, PMID: 27832756 PMC5105324

[ref48] Li X. F. Zhang X. J. Zhang C. Wang L. N. Li Y. R. Zhang Y.. (2018). Ulinastatin protects brain against cerebral ischemia/reperfusion injury through inhibiting MMP–9 and alleviating loss of ZO–1 and occludin proteins in mice. Exp. Neurol. 302, 68–74. doi: 10.1016/j.expneurol.2017.12.016, PMID: 29291404

[ref49] Liaudet L. Murthy K. G. Mabley J. G. Pacher P. Soriano F. G. Salzman A. L.. (2002). Comparison of inflammation, organ damage, and oxidant stress induced by *Salmonella enterica* Serovar Muenchen flagellin and serovar Enteritidis lipopolysaccharide. Infect. Immun. 70, 192–198. doi: 10.1128/IAI.70.1.192-198.2002, PMID: 11748182 PMC127621

[ref50] Liu G. H. Qu J. Shen X. (2008). NF-kappaB/p65 antagonizes Nrf2-ARE pathway by depriving CBP from Nrf2 and facilitating recruitment of HDAC3 to MafK. Biochim. Biophys. Acta 1783, 713–727. doi: 10.1016/j.bbamcr.2008.01.002, PMID: 18241676

[ref51] Luca M. Di Mauro M. Di Mauro M. Luca A. (2019). Gut microbiota in Alzheimer's disease, depression, and type 2 diabetes mellitus: the role of oxidative stress. Oxidative Med. Cell. Longev. 2019:4730539. doi: 10.1155/2019/4730539, PMID: 31178961 PMC6501164

[ref52] Magoč T. Salzberg S. L. (2011). FLASH: fast length adjustment of short reads to improve genome assemblies. Bioinformatics 27, 2957–2963. doi: 10.1093/bioinformatics/btr507, PMID: 21903629 PMC3198573

[ref53] Mazgaeen L. Gurung P. (2020). Recent advances in lipopolysaccharide recognition systems. Int. J. Mol. Sci. 21:379. doi: 10.3390/ijms21020379, PMID: 31936182 PMC7013859

[ref54] Meng X. Li S. Li Y. Gan R. Y. Li H. B. (2018). Gut Microbiota's relationship with liver disease and role in Hepatoprotection by dietary natural products and probiotics. Nutrients 10:1457. doi: 10.3390/nu10101457, PMID: 30297615 PMC6213031

[ref55] Parada Venegas D. De la Fuente M. K. Landskron G. González M. J. Quera R. Dijkstra G.. (2019). Short chain fatty acids (SCFAs)–mediated gut epithelial and immune regulation and its relevance for inflammatory bowel diseases. Front. Immunol. 10:277. doi: 10.3389/fimmu.2019.00277, PMID: 30915065 PMC6421268

[ref57] Pfeiler E. A. Azcarate-Peril M. A. Klaenhammer T. R. (2007). Characterization of a novel bile–inducible operon encoding a two–component regulatory system in *Lactobacillus acidophilus*. J. Bacteriol. 189, 4624–4634. doi: 10.1128/JB.00337-07, PMID: 17449631 PMC1913432

[ref58] Qiao J. Li H. Wang Z. Wang W. (2015). Effects of *Lactobacillus acidophilus* dietary supplementation on the performance, intestinal barrier function, rectal microflora and serum immune function in weaned piglets challenged with *Escherichia coli* lipopolysaccharide. Antonie Van Leeuwenhoek 107, 883–891. doi: 10.1007/s10482-015-0380-z, PMID: 25577203

[ref59] Quast C. Pruesse E. Yilmaz P. Gerken J. Schweer T. Yarza P.. (2013). The SILVA ribosomal RNA gene database project: improved data processing and web-based tools. Nucleic Acids Res. 41, D590–D596. doi: 10.1093/nar/gks1219, PMID: 23193283 PMC3531112

[ref60] Ruan Y. C. Wang Y. Da Silva N. Kim B. Diao R. Y. Hill E.. (2014). CFTR interacts with ZO–1 to regulate tight junction assembly and epithelial differentiation through the ZONAB pathway. J. Cell Sci. 127, 4396–4408. doi: 10.1242/jcs.148098, PMID: 25107366 PMC4197086

[ref61] Sassi A. Wang Y. Chassot A. Komarynets O. Roth I. Olivier V.. (2020). Interaction between epithelial Sodium Channel γ–subunit and Claudin–8 modulates Paracellular sodium permeability in renal collecting duct. J. Am. Soc. Nephrol. 31, 1009–1023. doi: 10.1681/ASN.2019080790, PMID: 32245797 PMC7217417

[ref62] Segain J. P. Raingeard de la Blétière D. Bourreille A. Leray V. Gervois N. Rosales C.. (2000). Butyrate inhibits inflammatory responses through NF–kappaB inhibition: implications for Crohn's disease. Gut 47, 397–403. doi: 10.1136/gut.47.3.397, PMID: 10940278 PMC1728045

[ref63] Shen J. Chen Y. J. Jia Y. W. Zhao W. Y. Chen G. H. Liu D. F.. (2017). Reverse effect of curcumin on CDDP-induced drug-resistance via Keap1/p62-Nrf2 signaling in A549/CDDP cell. Asian Pac J Trop Med 10, 1190–1196. doi: 10.1016/j.apjtm.2017.10.028, PMID: 29268977

[ref64] Shibuya N. Tanaka M. Yoshida M. Ogasawara Y. Togawa T. Ishii K.. (2009). 3-Mercaptopyruvate sulfurtransferase produces hydrogen sulfide and bound sulfane sulfur in the brain. Antioxid. Redox Signal. 11, 703–714. doi: 10.1089/ars.2008.2253, PMID: 18855522

[ref65] Silva-Islas C. A. Maldonado P. D. (2018). Canonical and non-canonical mechanisms of Nrf2 activation. Pharmacol. Res. 134, 92–99. doi: 10.1016/j.phrs.2018.06.013, PMID: 29913224

[ref66] Sommer F. Bäckhed F. (2013). The gut microbiota––masters of host development and physiology. Nat. Rev. Microbiol. 11, 227–238. doi: 10.1038/nrmicro2974, PMID: 23435359

[ref67] Splichalova A. Jenistova V. Splichalova Z. Splichal I. (2019). Colonization of preterm gnotobiotic piglets with probiotic *Lactobacillus rhamnosus* GG and its interference with *Salmonella* Typhimurium. Clin. Exp. Immunol. 195, 381–394. doi: 10.1111/cei.13236, PMID: 30422309 PMC6378394

[ref68] Stecher B. Berry D. Loy A. (2013). Colonization resistance and microbial ecophysiology: using gnotobiotic mouse models and single–cell technology to explore the intestinal jungle. FEMS Microbiol. Rev. 37, 793–829. doi: 10.1111/1574-6976.12024, PMID: 23662775

[ref69] Sun Z. Li H. Li Y. Qiao J. (2020). *Lactobacillus salivarius*, a potential probiotic to improve the health of LPS-challenged piglet intestine by alleviating inflammation as well as oxidative stress in a dose-dependent manner during weaning transition. Front. Vet. Sci. 7:547425. doi: 10.3389/fvets.2020.547425, PMID: 33392276 PMC7772421

[ref70] Sun X. Ou Z. Chen R. Niu X. Chen D. Kang R.. (2016). Activation of the p62–Keap1–NRF2 pathway protects against ferroptosis in hepatocellular carcinoma cells. Hepatology 63, 173–184. doi: 10.1002/hep.28251, PMID: 26403645 PMC4688087

[ref71] Suo H. Liu S. Li J. Ding Y. Wang H. Zhang Y.. (2018). *Lactobacillus paracasei* ssp. paracasei YBJ01 reduced d-galactose-induced oxidation in male Kuming mice. J. Dairy Sci. 101, 10664–10674. doi: 10.3168/jds.2018-14758, PMID: 30292551

[ref72] Suzuki T. Motohashi H. Yamamoto M. (2013). Toward clinical application of the Keap1-Nrf2 pathway. Trends Pharmacol. Sci. 34, 340–346. doi: 10.1016/j.tips.2013.04.005, PMID: 23664668

[ref73] Suzuki S. Toledo-Pereyra L. H. Rodriguez F. J. Cejalvo D. (1993). Neutrophil infiltration as an important factor in liver ischemia and reperfusion injury. Modulating effects of FK506 and cyclosporine. Transplantation 55, 1265–1272. doi: 10.1097/00007890-199306000-00011, PMID: 7685932

[ref74] Tang W. H. Kitai T. Hazen S. L. (2017). Gut microbiota in cardiovascular health and disease. Circ. Res. 120, 1183–1196. doi: 10.1161/CIRCRESAHA.117.309715, PMID: 28360349 PMC5390330

[ref75] Tang F. Xiong Y. Zhang H. Wu K. Xiang Y. Shao J. B.. (2016). Visual detection technique for efficient screening and isolation of Salmonella based on a novel enrichment assay using chromatography membrane. Eur. J. Clin. Microbiol. Infect. Dis. 35, 353–361. doi: 10.1007/s10096-015-2543-2, PMID: 26796551

[ref76] Trefts E. Gannon M. Wasserman D. H. (2017). The liver. Curr. Biol. 27, R1147–R1151. doi: 10.1016/j.cub.2017.09.019, PMID: 29112863 PMC5897118

[ref77] Valko M. Rhodes C. J. Moncol J. Izakovic M. Mazur M. (2006). Free radicals, metals and antioxidants in oxidative stress-induced cancer. Chem. Biol. Interact. 160, 1–40. doi: 10.1016/j.cbi.2005.12.009 16430879

[ref78] Wan M. L. Y. Forsythe S. J. El-Nezami H. (2019). Probiotics interaction with foodborne pathogens: a potential alternative to antibiotics and future challenges. Crit. Rev. Food Sci. 59, 3320–3333. doi: 10.1080/10408398.2018.1490885, PMID: 29993263

[ref79] Wang Q. Garrity G. M. Tiedje J. M. Cole J. R. (2007). Naive Bayesian classifier for rapid assignment of rRNA sequences into the new bacterial taxonomy. Appl. Environ. Microbiol. 73, 5261–5267. doi: 10.1128/AEM.00062-07, PMID: 17586664 PMC1950982

[ref80] Wang Z. Li B. Jiang H. Ma Y. Bao Y. Zhu X.. (2022). IL-8 exacerbates alcohol-induced fatty liver disease via the Akt/HIF-1α pathway in human IL-8-expressing mice. Cytokine 138:155402. doi: 10.1016/j.cyto.2020.155402, PMID: 33352397

[ref81] Wang L. Li L. Lv Y. Chen Q. Feng J. Zhao X. (2018). *Lactobacillus plantarum* restores intestinal permeability disrupted by Salmonella infection in newly-hatched chicks. Sci. Rep. 8:2229. doi: 10.1038/s41598-018-20752-z, PMID: 29396554 PMC5797085

[ref82] Wang H. Wang Q. Yang C. Guo M. Cui X. Jing Z.. (2022). *Bacteroides acidifaciens* in the gut plays a protective role against CD95-mediated liver injury. Gut Microbes 14:2027853. doi: 10.1080/19490976.2022.2027853, PMID: 35129072 PMC8820816

[ref83] Wu H. Wang Y. Zhang Y. Xu F. Chen J. Duan L.. (2020). Breaking the vicious loop between inflammation, oxidative stress and coagulation, a novel anti–thrombus insight of nattokinase by inhibiting LPS–induced inflammation and oxidative stress. Redox Biol. 32:101500. doi: 10.1016/j.redox.2020.101500, PMID: 32193146 PMC7078552

[ref84] Xia W. J. Xu M. L. Yu X. J. Du M. M. Li X. H. Yang T.. (2021). Antihypertensive effects of exercise involve reshaping of gut microbiota and improvement of gut–brain axis in spontaneously hypertensive rat. Gut Microbes 13, 1–24. doi: 10.1080/19490976.2020.1854642, PMID: 33382364 PMC7781639

[ref85] Xu H. Wang J. Cai J. Feng W. Wang Y. Liu Q.. (2019). Protective effect of *Lactobacillus rhamnosus* GG and its supernatant against myocardial dysfunction in obese mice exposed to intermittent hypoxia is associated with the activation of Nrf2 pathway. Int. J. Biol. Sci. 15, 2471–2483. doi: 10.7150/ijbs.36465, PMID: 31595164 PMC6775312

[ref86] Yang S. H. Yu L. H. Li L. Guo Y. Zhang Y. Long M.. (2018). Protective mechanism of Sulforaphane on cadmium-induced Sertoli cell injury in mice testis via Nrf2/ARE signaling pathway. Molecules 23:1774. doi: 10.3390/molecules23071774, PMID: 30029485 PMC6100605

[ref87] Zhang L. Gui S. Liang Z. Liu A. Chen Z. Tang Y.. (2019). *Musca domestica* Cecropin (mdc) alleviates *Salmonella* Typhimurium–induced colonic mucosal barrier impairment: associating with inflammatory and oxidative stress response, tight junction as well as intestinal Flora. Front. Microbiol. 10:522. doi: 10.3389/fmicb.2019.00522, PMID: 30930887 PMC6428779

[ref88] Zheng X. Qiu Y. Zhong W. Baxter S. Su M. Li Q.. (2013). A targeted metabolomic protocol for short–chain fatty acids and branched–chain amino acids. Metabolomics 9, 818–827. doi: 10.1007/s11306-013-0500-6, PMID: 23997757 PMC3756605

