# Supplementary material for: Lactobacillus acidophilus alleviate Salmonella enterica Serovar Typhimurium-induced murine inflammatory/oxidative responses via the p62-Keap1-Nrf2 signaling pathway and cecal microbiota
Source: Front Microbiol. 2025 Jan 16;15:1483705. doi: 10.3389/fmicb.2024.1483705 (PMC11781537; doi:10.3389/fmicb.2024.1483705)
Supplement: Supplementary file 1 [file Supplementary_file_1.docx]

Additional Document

# Supplementary Tables.

**TABLE S1.** Duodenal histopathology scoring criteria

| Score | Pathological manifestations |
| --- | --- |
| 0 | Normal intestinal villi |
| 1 | Separation of the villus epithelium of the from the lamina propria, and local increase of inflammatory cells in the lamina propria |
| 2 | Moderate separation of the villus epithelium from the lamina propria, and diffuse increase of inflammatory cells in the lamina propria |
| 3 | Heavy separation of the epithelium on both sides of the villi from the lamina propria, partial damage to the top of the villi, and accumulation of inflammatory cells under the endothelium |
| 4 | Breakage of villi, exposure of capillaries, increased cellular component of the intestinal lamina propria, diffusion of inflammatory cells under the endothelium |
| 5 | Structural incompleteness of the intestinal lamina propria, hemorrhage, ulceration, and massive accumulation of inflammatory cells |

**TABLE S2.** Liver pathology scoring criteria

| Score | Vacuolization | Lymphocytic infiltration | Necrosis |
| --- | --- | --- | --- |
| 0 | None | Normal | None |
| 1 | Minimal | Minimal | Single-cell necrosis |
| 2 | Mild (10%-30%) | Mild | Mild (10%-30%) |
| 3 | Moderate (31%-60%) | Moderate | Moderate (31%-60%) |
| 4 | Severe (>60%) | A large number of inflammatory cells exist in sheets | Severe (>60%) |

## Supplementary Figure


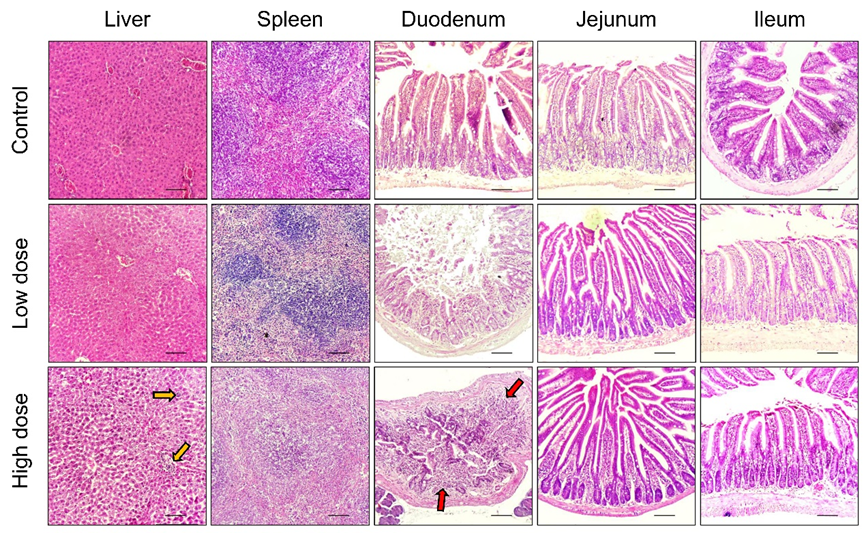


**FIGURE. S1.** Histological section results of mouse tissues. Use saline (Control), Low dose ST (3.2 × 10^7^ CFU/mL) and High dose ST (3.2 × 10^8^ CFU/mL). The mice were sacrificed 24 h after gavage, and the liver, spleen, duodenum, jejunum and ileum of the mice were collected for routine paraffin sections and H&E staining to observe the results.
